# Prenatal opioid exposure significantly impacts placental protein kinase C (PKC) and drug transporters, leading to drug resistance and neonatal opioid withdrawal syndrome

**DOI:** 10.3389/fnins.2024.1442915

**Published:** 2024-08-19

**Authors:** Uppala Radhakrishna, Rupa Radhakrishnan, Lavanya V. Uppala, Srinivas B. Muvvala, Jignesh Prajapati, Rakesh M. Rawal, Ray O. Bahado-Singh, Senthilkumar Sadhasivam

**Affiliations:** ^1^Department of Anesthesiology and Perioperative Medicine, University of Pittsburgh, Pittsburgh, PA, United States; ^2^Department of Obstetrics and Gynecology, Corewell Health William Beaumont University Hospital, Royal Oak, MI, United States; ^3^Department of Radiology and Imaging Sciences, Indiana University School of Medicine, Indianapolis, IN, United States; ^4^College of Information Science & Technology, the University of Nebraska at Omaha, Peter Kiewit Institute, Omaha, NE, United States; ^5^Department of Psychiatry, Yale School of Medicine, New Haven, CT, United States; ^6^Department of Biochemistry & Forensic Sciences, Gujarat University, Ahmedabad, India; ^7^Department of Medical Biotechnology, Gujarat Biotechnology University, Gandhinagar, Gujarat, India

**Keywords:** drug transporters, protein kinases C, biomarker, opioid use, neonatal opioid withdrawal syndrome SLC transporters ABC transporters, bile secretion, pancreatic secretion, insulin resistance

## Abstract

**Background:**

Neonatal Opioid Withdrawal Syndrome (NOWS) is a consequence of in-utero exposure to prenatal maternal opioids, resulting in the manifestation of symptoms like irritability, feeding problems, tremors, and withdrawal signs. Opioid use disorder (OUD) during pregnancy can profoundly impact both mother and fetus, disrupting fetal brain neurotransmission and potentially leading to long-term neurological, behavioral, and vision issues, and increased infant mortality. Drug resistance complicates OUD and NOWS treatment, with protein kinase regulation of drug transporters not fully understood.

**Methods:**

DNA methylation levels of ATP-binding cassette (ABC) and solute carrier (SLC) drug transporters, along with protein kinase C (PKC) genes, were assessed in 96 placental samples using the Illumina Infinium MethylationEPIC array (850K). Samples were collected from three distinct groups: 32 mothers with infants prenatally exposed to opioids who needed pharmacological intervention for NOWS, 32 mothers with prenatally opioid-exposed infants who did not necessitate NOWS treatment, and 32 mothers who were not exposed to opioids during pregnancy.

**Results:**

We identified 69 significantly differentially methylated SLCs, with 24 hypermethylated and 34 hypomethylated, and 11 exhibiting both types of methylation changes including *SLC13A3, SLC15A2, SLC16A11, SLC16A3, SLC19A2*, and *SLC26A1*. We identified methylation changes in 11 ABC drug transporters *(ABCA1, ABCA12, ABCA2, ABCB10, ABCB5, ABCC12, ABCC2, ABCC9, ABCE1, ABCC7, ABCB3*): 3 showed hypermethylation, 3 hypomethylation, and 5 exhibited both. Additionally, 7 PKC family genes (*PRKCQ, PRKAA1, PRKCA, PRKCB, PRKCH, PRKCI*, and *PRKCZ*) showed methylation changes. These genes are associated with 13 pathways involved in NOWS, including ABC transporters, bile secretion, pancreatic secretion, insulin resistance, glutamatergic synapse, and gastric acid secretion.

**Conclusion:**

We report epigenetic changes in PKC-related regulation of drug transporters, which could improve our understanding of clinical outcomes like drug resistance, pharmacokinetics, drug-drug interactions, and drug toxicity, leading to maternal relapse and severe NOWS. Novel drugs targeting PKC pathways and transporters may improve treatment outcomes for OUD in pregnancy and NOWS.

## Introduction

Opioid use disorder (OUD) represents a significant global health challenge. Maternal opioid misuse during pregnancy can result in Neonatal Opioid Withdrawal Syndrome (NOWS), which poses severe risks to newborns, including irritability, feeding difficulties, tremors, and withdrawal symptoms. These effects may extend into later life, impacting neurodevelopment, behavior, mental health, and potentially vision-related issues (Anbalagan and Mendez, 2023). Genetic and epigenetic variations in opioid receptors, metabolic enzymes, regulatory proteins, and transporters significantly influence susceptibility to NOWS (Metpally et al., 2019; Radhakrishna et al., 2021a,b, 2023a,b). Despite effective treatments for OUD and NOWS, drug resistance persists as a significant challenge (Wang X. et al., 2019). The roles of transporter proteins and signal transduction enzymes in drug resistance related to OUD and NOWS are not well understood.

Transporters are essential for pharmacokinetics and pharmacodynamics, impacting drug interactions, adverse effects, and body homeostasis by facilitating the transfer of ions, amino acids, sugars, and drugs across cell membranes (Zhang, 2018; Peng et al., 2020; Carbo and Rodriguez, 2023). Drug resistance arises from various mechanisms, with drug transporters and metabolizers significantly impacting drug efficacy (Mansoori et al., 2017; Kawano et al., 2022). Dysregulation in these genes can alter neurotransmitter levels and signaling pathways, contributing to opioid dependence and addiction (Mistry et al., 2014). Transporters, commonly situated on the plasma membrane, can be divided into solute carriers (SLC) and ATP-binding cassette (ABC) transporters. Both solute carrier (SLC) and ATP-binding cassette (ABC) drug transporters can be regulated by PKCs-related signaling pathways (Mayati et al., 2017; Puris et al., 2023). The SLC transporters include over 400 members across 52 families (Hediger et al., 2013; Baril et al., 2023). Most SLC transporters are responsible for the uptake of small molecules (including nutrients and xenobiotics), but a few SLCs act as both influx and efflux transporters. They are expressed in organs like the intestines, liver, and kidneys, which are involved in drug absorption, metabolism, and elimination (Brecht et al., 2020).

ABC transporters encompass a diverse group of 48 known proteins categorized into seven primary types: ABCA, ABCB, ABCC, ABCD, ABCE, ABCF, and ABCG (Dean et al., 2001). ABC transporters use ATP hydrolysis to move a variety of substances across cell membranes. ABC transporters in the human placenta transport endogenous compounds and protect the fetus from exogenous substances like therapeutic agents, drugs of abuse, and other xenobiotics (Joshi et al., 2016). These transporters are linked to diseases like cystic fibrosis (*ABCC7/CFTR*), Tangier disease, cardiovascular disease (*ABCA1*), retinitis pigmentosa (*ABCA4*), and more (Vasiliou et al., 2009).

Protein Kinase C (PKC) is a family of serine/threonine kinases that play crucial roles in the proliferation, differentiation, survival, migration, invasion, apoptosis, and anticancer drug resistance of cancer cells (Kawano et al., 2022). Dysregulated PKC can cause abnormal phosphorylation and misregulation of SLC and ABC transporters, disrupting their activity and gene expression. This affects drug absorption, potentially leading to resistance or toxicity, and interferes with transporter trafficking and protein interactions, further disrupting cellular transport processes (Alves et al., 2022).

Previous reports highlight the significant roles of transporter genes such as *SLC6A3 (DAT1)*, which regulates dopamine reuptake affecting reward pathways crucial for addiction development, and *SLC6A4 (SERT)*, which controls serotonin levels impacting mood and emotional stability, in neurotransmitter regulation linked to addiction development and responses to opioids (Grover et al., 2020; Yuferov et al., 2021). PKC-related signaling pathways can regulate both SLC and ABC drug transporters. In the context of OUD, PKCs play a significant role by influencing addiction, tolerance, dependence, withdrawal, and drug-seeking behavior (Lee and Messing, 2008).

Our study used genome-wide methylation analysis to explore whether epigenetic modifications of placental PKC and drug transporters in infants exposed to prenatal opioids could predict NOWS. We found significant methylation changes in multiple PKCs and drug transporter genes involved in NOWS development. Targeting these transporters and PKC could lead to new therapeutic approaches for treating opioid addiction and managing NOWS.

## Materials and methods

The research study received approval from the Institutional Review Board of Beaumont Health System, Royal Oak, MI, USA (HIC#: 2019-086). Pregnant women were identified retrospectively through chart review at William Beaumont Hospital, Royal Oak, MI. Informed consent was not required for this study as it solely involved the collection of discarded placental tissues from the subjects, along with obtaining limited de-identified, data from the hospital medical records. We collected demographic and clinical-pathological data, including age, sex, ethnicity, gestational age, and history of drug exposure (Radhakrishna et al., 2021b). Patients were diagnosed according to the assessment criteria outlined in the Diagnostic and Statistical Manual of Mental Disorders, Fifth Edition (DSM-5) (Hasin et al., 2013).

The sample details and methodology have been documented in our prior publication (Radhakrishna et al., 2021b). To summarize, ninety-six formalin-fixed, paraffin-embedded (FFPE) placental tissue biopsies were collected and processed. These tissue samples were categorized into three groups: Group 1 comprised 32 newborns prenatally exposed to opioids requiring treatment for Neonatal Opioid Withdrawal Syndrome (NOWS) (+Opioids/+NOWS), Group 2 included 32 newborns prenatally exposed to opioids not requiring treatment for NOWS (+Opioids/-NOWS), and Group-3 served as the control group consisting of newborns with no prenatal opioid exposure and no NOWS (-Opioids/-NOWS, control). The mean gestational age at delivery (in weeks) was 37.94 (SD = 3.16) for Group 1, 37.49 (SD = 2.96) for Group 2, and 38.09 (SD = 3.37) for Group 3. NOWS diagnosis (P96.1) was determined by neonatologists based on clinical criteria. Infants born to mothers with a history of opioid or illicit drug use were observed in the inpatient unit for 4–5 days to detect signs of NOWS. Scoring was conducted using the Finnegan Neonatal Abstinence Scoring Tool (FNAST). Postpartum nurses and/or NICU nurses conducted the scoring process. If the scores indicated a need for pharmacologic treatment according to set criteria, the infant was moved to the NICU for continued monitoring, scoring, and treatment. Parental involvement was encouraged to enhance non-pharmacologic interventions as the primary approach before and during treatment, regardless of whether the infant received pharmacologic treatment. The initiation of pharmacologic management with morphine was determined using the Finnegan Neonatal Abstinence Scoring Tool (FNAST). The analysis comprised four comparisons: I. (+Opioids/+NOWS) vs. (+Opioids/-NOWS); II. (+Opioids/+NOWS) + (+Opioids/-NOWS) vs. (-Opioids/-NOWS, control); III. (+Opioids/+NOWS) vs. (-Opioids/-NOWS, control); IV. (+Opioids/-NOWS) vs. (-Opioids/-NOWS, control), calculated for each unique differentially methylated CpG locus.

### Methylation analysis

All participant mothers with OUD were of European-American ancestry. Placental specimens were collected from the maternal side, approximately 2 cm from the site of umbilical cord insertion. Generally, eight to ten 10 mm curls of formalin-fixed paraffin-embedded (FFPE) placental tissue from each block were used for DNA preparation. The extensive discussion of Illumina Infinium MethylationEPIC array BeadChip (850K) assay (Illumina, Inc., San Diego, CA, USA) has been previously referenced (Radhakrishna et al., 2021b). These state-of-the-art arrays boast coverage of over 850,000 CpG sites across the genome, offering unparalleled single-nucleotide precision. We obtained data on differentially methylated CG dinucleotides from previously unpublished DNA methylation datasets concerning SLC transporters, ABC drug transporters, and PKC family genes (Radhakrishna et al., 2021b). The information on SLC transporters was sourced from https://slc.bioparadigms.org/, ABC transporters from http://www.genenames.org, and the PKC gene family was obtained from https://rgd.mcw.edu/rgdweb/homepage/.

### Statistical and bioinformatic analysis

Before analysis, CpG-probes with missing ß-values were excluded. Differential methylation was evaluated by comparing ß-values for cytosines at each CpG locus between NOWS and controls. Probes linked to sex chromosomes, non-specific probes, and those targeting CpG sites within 10 bp of SNPs were removed to mitigate confounding factors. SNPs with a minor allele frequency ≤ 0.05 were considered for further analysis (Liu et al., 2013; Wilhelm-Benartzi et al., 2013; Zhang et al., 2013).

The *p*-value for methylation differences between the case and control groups at each locus was computed as outlined previously (Vishweswaraiah et al., 2019; Radhakrishna et al., 2021b). CpG sites showing significant differential methylation between NOWS, and controls were identified using predefined cutoff criteria of FDR *p* < 0.05 and retained for further analysis. Raw and FDR-adjusted *p*-values for multiple testing (using the Benjamini-Hochberg test) were calculated. The area under the receiver operating characteristic curve (AUC-ROC) for combinations of loci was determined using the ‘ROCR’ package (v3.5.0) in the ‘R’ program, based on methylation levels at the most significantly differentially methylated CpG loci.

### Network interaction analysis using STRING

Protein-protein interaction analysis was conducted using the STRING database (version 12.0), available at http://string-db.org, following the identification of differently methylated genes with an FDR *p*-value < 0.05. The database compiles known and predicted protein-protein interactions, including both physical and functional associations. For the analysis, all interaction sources were utilized: text mining from scientific literature, experimental data, aggregated information from curated databases, co-expression data, genomic context predictions (neighbourhood), evidence from gene fusion events, and phylogenetic tree-based co-occurrence. To ensure the interactions were significant while maintaining a comprehensive dataset, a medium confidence score threshold of 0.400 was applied. This method aimed to create a balanced protein interaction network, minimizing the inclusion of false positives yet allowing for the discovery of potentially relevant associations that may elucidate the complex mechanisms underlying NOWS.

### Gene ontology (GO) and KEGG pathway analyses

We conducted gene ontology (GO) and Kyoto Encyclopedia of Genes and Genomes (KEGG) pathway analyses on the same pool of differentially methylated genes, identified with an FDR *p*-value < 0.05. This was aimed at unveiling their biological relevance and participation in dysregulated signaling pathways associated with drug resistance. For GO analysis, the identified genes were categorized into three main ontologies: Biological Process (BP), Cellular Component (CC), and Molecular Function (MF). This classification provided insights into the functional roles of the genes, their cellular localization, and the molecular activities they may influence. KEGG analysis was conducted to integrate the differently methylated genes into known genetic pathways, providing an understanding of how alterations in gene methylation could affect specific biological pathways and processes. This integration helps to identify which pathways are potentially altered in the context of NOWS. Both GO and KEGG analyses utilized ‘clusterProfiler’, an R package designed for statistical analysis and visualization of functional profiles for genes and gene clusters. It facilitates the comparison of biological themes among gene clusters, enhancing the interpretation of high-throughput genomics data.

### Heatmaps

Differential methylation patterns of CpG sites, especially those associated with dysregulated transporters such as ABC genes, SLC genes, and PKC genes related to pain, were used to generate a heatmap using the ComplexHeatmap (v1.6.0) package in the R environment (v3.2.2). Sample hierarchical clustering was conducted using Ward distance (Gu, 2015).

## Results

### Analysis of transporters & PKCs in NOWS

**FIGURE 1:**
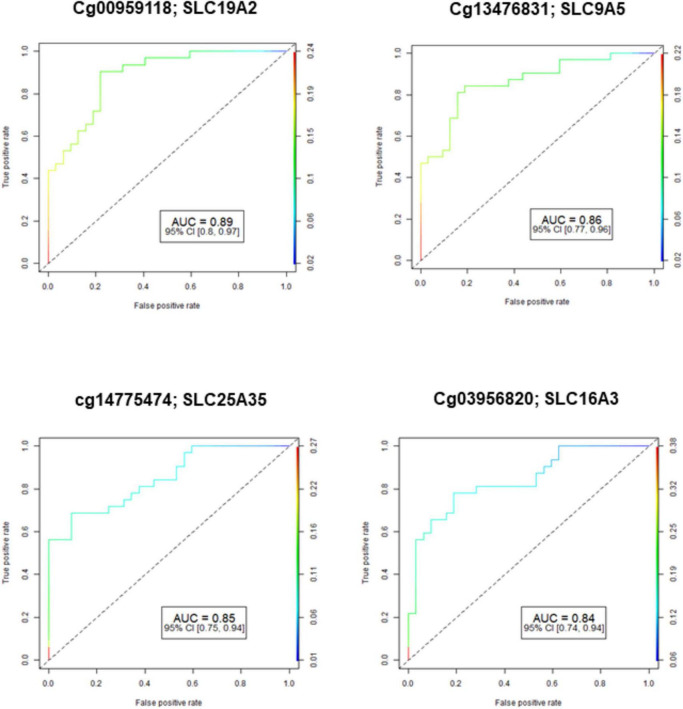
Receiver operating characteristic (ROC) curve analysis of methylated CpGs in ABC, SLC, and PKC genes (FDR-p ≤ 0.05) that had good diagnostic accuracy with CpGs AUC ≥ 0.80-0.89). AUC: Area Under the Receiver Operating Characteristics Curve; 95% CI: 95% Confidence Interval. Confidence intervals (CI) in parentheses show bounds.

The demographic characteristics of both NOWS and control groups were examined, indicating no significant disparities. This data has been previously published (Radhakrishna et al., 2021a). Figure 1 shows the Receiver Operating Characteristic (ROC) curve analysis of four significantly differently methylated CpGs in ABC, SLC, and PKC genes (FDR *p* ≤ 0.05). The results of four different analyses showed that Analysis I identified 8 dysregulated ABC genes, 38 SLC genes, and 2 PKC genes when comparing individuals with (+Opioids/+NOWS) to those with (+Opioids/-NOWS) (Supplementary Table 1). Analysis II compared individuals with (+Opioids/+NOWS) and (+Opioids/-NOWS) against those with (-Opioids/-NOWS, control), identifying 2 ABC genes, 15 SLC genes, and 4 PKC genes (Supplementary Table 2). Analysis III showed differential regulation of 1 ABC gene, 29 SLC genes, and 1 PKC gene in individuals with (+Opioids/+NOWS) compared to those with (-Opioids/-NOWS, control) (Supplementary Table 3). Analysis IV revealed differential regulation of 6 ABC genes, 18 SLC genes, and 5 PKC genes in individuals with (+Opioids/-NOWS) compared to those with (-Opioids/-NOWS, control) (Supplementary Table 4).

Across all four analyses, multiple genes were identified, totaling 87, showing significant methylation changes in genes linked to drug transporters and PKCs. Among them, 69 SLC transporters exhibit various methylation patterns: 24 are hypermethylated, 34 are hypomethylated, and 11 display both hypo- and hypermethylation. We observed 11 ABC drug transporters: *ABCA1, ABCA12, ABCA2, ABCB10, ABCB5, ABCC12, ABCC2, ABCC9, ABCE1, CFTR (ABCC7*), and *TAP2 (ABCB3*) that were differentially methylated. Among these, *ABCA2, ABCC12*, and *ABCE1* were found to have hypomethylation, while *ABCC2, ABCC9*, and *TAP2 (ABCB3*) exhibited hypermethylation. Notably, *ABCA1, ABCA12, ABCB10, ABCB5*, and *CFTR* (*ABCC7*) genes showed both hypo- and hypermethylation tendencies. Furthermore, variations in methylation were noted in 7 genes of the protein kinase C family, including hypomethylated genes *PRKAA1, PRKCB*, and *PRKCH*, hypermethylated gene *PRKCA*, and genes *PRKCI, PRKCQ*, and *PRKCZ* displaying both hypo- and hypermethylation (Table 1).

**TABLE 1:** A comprehensive detail of CpG targets that exhibit significant differential methylation in NOWS.

Target ID	Genes	Location	*p*-Value	FDR *p*-Value	% Methylation			AUC	CI		Gene detail
					Cases	Control	Change		Lower	Upper	
**cg10434274**	**ABCA1**	9q31.1	4.14763E-09	0.003587703	60.83	68.83	−8.00	0.75	0.63	0.87	ABC
**cg14299235**	**ABCA1**	9q31.1	5.50932E-10	0.000476556	67.75	59.84	7.91	0.68	0.55	0.81	ABC
cg00972111	ABCA12	2q35	4.72015E-14	4.08293E-08	78.32	70.24	8.08	0.73	0.61	0.85	ABC
cg02820283	ABCA2	9q34.3	2.81873E-08	0.02438205	72.63	78.14	−5.51	0.71	0.60	0.83	ABC
**cg21045171**	**ABCB10**	1q42.13	2.51239E-11	2.17322E-05	85.30	79.30	6.00	0.79	0.68	0.90	ABC
**cg21045171**	**ABCB10**	1q42.13	2.66472E-08	0.023049813	78.01	83.60	−5.59	0.75	0.64	0.87	ABC
**cg08888968**	**ABCB5**	7p21.1	2.90402E-16	2.51198E-10	68.08	77.76	−9.69	0.76	0.64	0.88	ABC
**cg08888968**	**ABCB5**	7p21.1	9.25029E-39	8.0015E-33	80.18	70.50	9.68	0.75	0.63	0.87	ABC
cg21723907	ABCC12	16q12.1	8.67446E-09	0.007503411	74.56	80.83	−6.28	0.78	0.67	0.89	ABC
cg01614760	ABCC2	10q24.2	2.00132E-08	0.017311439	43.41	35.80	7.60	0.74	0.62	0.86	ABC
cg01909678	ABCC9	12p12.1	3.14029E-14	2.71635E-08	76.39	67.95	8.44	0.78	0.67	0.89	ABC
cg26690672	ABCE1	4q31.21	2.09819E-12	1.81494E-06	63.82	72.88	−9.06	0.76	0.64	0.88	ABC
**cg26635219**	**ABCC7/CFTR**	7q31.2	9.66969E-39	8.36428E-33	24.59	14.19	10.40	0.76	0.64	0.88	ABC
**cg22533025**	**ABCC7/CFTR**	7q31.2	1.97867E-11	1.71155E-05	72.00	79.58	−7.59	0.73	0.61	0.85	ABC
cg00720839	ABCB3/TAP2	6p21.32	6.28153E-09	0.005433526	80.38	74.32	6.06	0.64	0.50	0.78	ABC
cg22826226	SLC11A2	12q13.12	7.94208E-09	0.006869897	80.63	74.65	5.98	0.76	0.65	0.88	SLC
cg10439765	SLC12A5	20q13.12	2.61284E-08	0.022601057	15.53	21.81	−6.27	0.70	0.57	0.82	SLC
cg18756954	SLC12A7	5p15.33	2.03828E-08	0.017631088	72.18	78.71	−6.53	0.74	0.62	0.86	SLC
cg15062310	SLC12A9	7q22.1	5.60196E-08	0.048456948	14.05	9.28	4.77	0.78	0.66	0.89	SLC
cg09192862	SLC13A3	20q13.12	2.0586E-08	0.017806874	79.71	85.13	−5.42	0.84	0.74	0.94	SLC
cg02131891	SLC15A2	3q13.33	2.47989E-09	0.002145104	78.88	84.68	−5.80	0.83	0.73	0.94	SLC
cg15639045	SLC16A11	17p13.1	3.49989E-08	0.03027406	55.79	63.69	−7.90	0.76	0.65	0.88	SLC
**cg03956820**	**SLC16A3**	17q25.3	4.63951E-09	0.004013176	17.56	12.01	5.55	0.84	0.74	0.94	SLC
**cg08296680**	**SLC16A3**	17q25.3	3.54873E-09	0.00306965	44.17	51.88	−7.72	0.67	0.55	0.79	SLC
**cg13460167**	**SLC17A5**	6q13	9.83035E-10	0.000850325	56.82	65.43	−8.61	0.78	0.67	0.90	SLC
**cg24865549**	**SLC17A5**	6q13	2.09993E-08	0.018164393	85.53	80.50	5.04	0.72	0.60	0.85	SLC
cg02624701	SLC17A7	19q13.33	1.26807E-08	0.010968811	30.60	23.62	6.98	0.71	0.58	0.84	SLC
**cg12285003**	**SLC17A9**	20q13.33	3.10992E-09	0.002690084	71.71	78.63	−6.91	0.83	0.73	0.93	SLC
**cg12285003**	**SLC17A9**	20q13.33	1.08447E-08	0.00938070	73.03	78.63	−5.60	0.78	0.67	0.88	SLC
cg00959118	SLC19A2	1q24.2	7.31371E-10	0.000632636	17.10	11.39	5.71	0.89	0.80	0.97	SLC
cg09726804	SLC19A3	2q36.3	8.28861E-09	0.007169646	73.34	66.44	6.90	0.73	0.61	0.86	SLC
**cg03989758**	**SLC1A3**	5p13.2	1.52389E-11	1.31816E-05	69.13	77.23	−8.10	0.76	0.65	0.88	SLC
**cg06960901**	**SLC1A3**	5p13.2	4.06686E-09	0.003517831	27.60	20.80	6.80	0.70	0.58	0.83	SLC
cg05414613	SLC1A7	1p32.3	1.97336E-11	1.70696E-05	62.49	71.39	−8.90	0.82	0.72	0.93	SLC
cg17949403	SLC22A23	6p25.2	1.62173E-08	0.014027981	63.91	56.38	7.53	0.70	0.57	0.83	SLC
cg11713788	SLC22A23	6p25.2	1.14405E-09	0.00098960	15.53	21.56	−6.03	0.55	0.43	0.68	SLC
cg01739295	SLC22A3	6q25.3	3.72641E-09	0.003223343	34.96	27.49	7.47	0.73	0.60	0.85	SLC
**cg17978727**	**SLC23A2**	20p13	3.2701E-08	0.028286374	35.05	27.91	7.14	0.86	0.77	0.95	SLC
**cg13282929**	**SLC23A2**	20p13	1.05376E-11	9.11502E-06	38.36	48.43	−10.07	0.78	0.66	0.89	SLC
cg13223777	SLC24A5	15q21.1	1.26703E-14	1.09598E-08	72.94	81.33	−8.39	0.85	0.76	0.95	SLC
cg12030923	SLC25A13	7q21.3	3.85551E-10	0.000333502	64.94	72.99	−8.04	0.73	0.61	0.85	SLC
cg14710071	SLC25A16	10q21.3	4.75308E-08	0.041114139	80.54	85.65	−5.11	0.78	0.66	0.89	SLC
cg23045610	SLC25A24	1p13.3	1.71658E-09	0.001484844	64.20	56.25	7.95	0.77	0.65	0.88	SLC
**cg00298230**	**SLC25A26**	3p14.1	4.97067E-11	4.29963E-05	79.20	85.45	−6.25	0.85	0.75	0.94	SLC
**cg10343071**	**SLC25A26**	3p14.1	5.46218E-11	4.72479E-05	73.11	65.32	7.79	0.78	0.66	0.89	SLC
**cg25217269**	**SLC25A27**	6p12.3	1.95076E-11	1.68741E-05	29.90	38.17	−8.27	0.73	0.62	0.84	SLC
**cg25217269**	**SLC25A27**	6p12.3	2.82907E-09	0.002447147	29.73	38.17	−8.44	0.71	0.59	0.84	SLC
cg27155504	SLC25A3	12q23.1	3.46705E-09	0.002999001	80.86	74.78	6.08	0.77	0.65	0.88	SLC
cg14775474	SLC25A35	17p13.1	1.44284E-08	0.01248054	11.75	7.23	4.52	0.85	0.75	0.94	SLC
cg18270394	SLC25A36	3q23	1.46857E-11	1.27031E-05	77.30	84.04	−6.74	0.75	0.63	0.87	SLC
cg03084648	SLC25A37	8p21.2	5.46944E-09	0.00473107	77.37	83.36	−5.99	0.77	0.65	0.88	SLC
cg16405055	SLC25A44	1q22	1.02268E-10	8.84615E-05	7.03	11.86	−4.83	0.65	0.53	0.77	SLC
cg09153458	SLC26A1	4p16.3	4.46293E-13	3.86043E-07	55.56	45.90	9.66	0.77	0.65	0.88	SLC
**cg26303603**	**SLC26A2**	5q32	2.80194E-11	2.42368E-05	59.10	50.17	8.93	0.78	0.67	0.89	SLC
**cg26303603**	**SLC26A2**	5q32	1.71203E-08	0.014809033	47.20	55.60	−8.40	0.75	0.63	0.87	SLC
**cg12588047**	**SLC28A3**	9q21.32-q21.33	2.14091E-14	1.85189E-08	77.39	69.01	8.38	0.75	0.63	0.87	SLC
**cg12588047**	**SLC28A3**	9q21.32-q21.33	8.78757E-10	0.000760125	67.46	75.07	−7.60	0.71	0.59	0.84	SLC
cg00309135	SLC2A12	6q23.2	1.74595E-09	0.001510246	71.49	78.46	−6.97	0.77	0.66	0.89	SLC
**cg07645864**	**SLC2A13**	12q12	4.80512E-08	0.041564282	81.05	75.43	5.62	0.73	0.61	0.86	SLC
**cg07645864**	**SLC2A13**	12q12	4.7902E-11	4.14352E-05	71.55	79.07	−7.53	0.73	0.61	0.85	SLC
cg19132526	SLC2A2	3q26.2	8.64142E-12	7.47483E-06	51.94	61.80	−9.86	0.78	0.67	0.89	SLC
cg20566657	SLC2A9	4p16.1	3.53496E-09	0.003057739	64.98	72.69	−7.71	0.82	0.72	0.92	SLC
cg08789022	SLC30A3	2p23.3	2.09826E-38	1.815E-32	24.84	15.24	9.60	0.75	0.63	0.87	SLC
**cg16989032**	**SLC30A4**	15q21.1	3.04023E-12	2.6298E-06	61.32	70.61	−9.29	0.76	0.64	0.88	SLC
**cg16989032**	**SLC30A4**	15q21.1	1.74464E-11	1.50912E-05	73.48	65.55	7.93	0.70	0.57	0.83	SLC
cg02530515	SLC30A7	1p21.2	1.18431E-08	0.010244289	69.42	76.32	−6.90	0.76	0.65	0.88	SLC
cg11178666	SLC33A1	3q25.31	2.61279E-09	0.002260067	56.99	65.38	−8.40	0.76	0.64	0.87	SLC
cg02272859	SLC34A2	4p15.2	1.42711E-08	0.012344479	58.59	66.60	−8.01	0.78	0.67	0.89	SLC
cg16584327	SLC35B4	7q33	3.26095E-11	2.82072E-05	69.14	77.07	−7.93	0.75	0.63	0.87	SLC
cg26453171	SLC35F2	11q22.3	4.69575E-12	4.06182E-06	13.57	20.97	−7.40	0.77	0.65	0.88	SLC
cg14469376	SLC37A2	11q24.2	4.6003E-09	0.003979261	9.77	5.46	4.31	0.78	0.67	0.89	SLC
cg02765475	SLC38A9	5q11.2	4.08508E-09	0.003533594	75.04	81.37	−6.33	0.75	0.64	0.87	SLC
cg00003999	SLC39A10	2q32.3	6.45175E-09	0.005580765	79.37	73.18	6.20	0.74	0.61	0.86	SLC
cg14228592	SLC39A4	8q24.3	8.63426E-11	7.46864E-05	21.89	15.16	6.74	0.70	0.57	0.83	SLC
cg17926678	SLC39A9	14q24.1	1.62919E-38	1.40925E-32	34.99	24.49	10.50	0.77	0.65	0.88	SLC
**cg25287207**	**SLC41A2**	12q23.3	2.1923E-09	0.001896343	71.73	78.63	−6.90	0.80	0.69	0.91	SLC
**cg25287207**	**SLC41A2**	12q23.3	6.23886E-11	5.39661E-05	80.33	73.56	6.77	0.79	0.68	0.90	SLC
cg07291744	SLC43A1	11q12.1	2.60106E-39	2.24991E-33	56.96	45.99	10.97	0.58	0.44	0.72	SLC
cg19272348	SLC43A2	17p13.3	2.95112E-10	0.000255272	73.67	80.66	−6.99	0.76	0.65	0.88	SLC
cg22521553	SLC44A1	9q31.1-q31.2	1.22738E-12	1.06169E-06	73.51	65.18	8.33	0.71	0.58	0.83	SLC
cg02426178	SLC44A2	19p13.2	2.01983E-08	0.01747157	80.20	85.49	−5.29	0.70	0.57	0.83	SLC
cg08203794	SLC45A4	8q24.3	2.12648E-11	1.8394E-05	83.88	77.62	6.26	0.81	0.70	0.92	SLC
cg17156227	SLC4A11	20p13	8.58965E-12	7.43005E-06	65.62	74.28	−8.66	0.79	0.67	0.90	SLC
cg09988421	SLC4A2	7q36.1	1.9444E-09	0.001681904	26.37	19.51	6.86	0.78	0.67	0.90	SLC
cg20078681	SLC4A3	2q35	1.86189E-08	0.016105309	84.71	89.24	−4.53	0.85	0.76	0.95	SLC
cg13250541	SLC5A6	2p23.3	2.56509E-14	2.2188E-08	13.99	7.73	6.26	0.85	0.75	0.95	SLC
cg26758670	SLC6A12	12p13.33	4.31442E-10	0.000373197	18.40	12.37	6.03	0.79	0.68	0.90	SLC
cg04394707	SLC6A15	12q21.31	2.35189E-08	0.020343858	60.54	68.20	−7.66	0.72	0.60	0.85	SLC
cg16321159	SLC6A17	1p13.3	3.57318E-08	0.030907997	81.60	86.58	−4.98	0.76	0.65	0.88	SLC
cg17277001	SLC6A18	5p15.33	8.83477E-09	0.007642076	28.79	36.89	−8.10	0.71	0.58	0.84	SLC
cg26339753	SLC6A20	3p21.31	1.3871E-09	0.001199845	58.65	67.14	−8.49	0.73	0.61	0.86	SLC
cg06617455	SLC6A6	3p25.1	1.60204E-08	0.013857662	51.42	59.71	−8.29	0.76	0.65	0.88	SLC
cg22367705	SLC9A1	1p36.11	1.4567E-14	1.26004E-08	18.10	11.05	7.05	0.78	0.66	0.89	SLC
cg13476831	SLC9A5	16q22.1	1.00782E-09	0.000871768	15.73	10.21	5.52	0.88	0.79	0.96	SLC
cg25268697	SLCO1B3	12p12.2	6.83856E-12	5.91536E-06	35.62	44.35	−8.74	0.66	0.54	0.78	SLC
cg22822824	SLCO2A1	3q22.1-q22.2	3.31677E-08	0.02869003	6.18	10.14	−3.96	0.61	0.48	0.73	SLC
cg12878682	SLCO5A1	8q13.3	2.47908E-12	2.14441E-06	68.47	76.89	−8.42	0.74	0.62	0.86	SLC
cg26294217	PRKAA1	5p13.1	6.80901E-09	0.005889795	5.54	10.08	−4.54	0.76	0.65	0.88	PKC
cg11899080	PRKCA	17q24.2	5.88738E-39	5.09258E-33	65.29	55.15	10.15	0.82	0.71	0.92	PKC
cg13127598	PRKCB	16p12.2-p12.1	1.76442E-08	0.01526223	18.23	24.17	−5.94	0.57	0.45	0.70	PKC
cg18417061	PRKCH	14q23.1	6.21097E-10	0.00053725	76.05	81.62	−5.57	0.81	0.71	0.91	PKC
**cg05878107**	**PRKCI**	3q26.2	7.36142E-39	6.36763E-33	72.02	62.10	9.92	0.77	0.66	0.89	PKC
**cg08532220**	**PRKCI**	3q26.2	2.61116E-10	0.000225865	57.36	66.20	−8.84	0.74	0.62	0.86	PKC
**cg00105154**	**PRKCQ**	10p15.1	9.19432E-14	7.95308E-08	21.46	14.05	7.42	0.75	0.63	0.87	PKC
**cg00105154**	**PRKCQ**	10p15.1	5.72985E-13	4.95632E-07	14.86	22.82	−7.97	0.70	0.57	0.83	PKC
**cg16269144**	**PRKCZ**	1p36.33	3.26129E-10	0.000282101	80.02	73.44	6.58	0.77	0.65	0.89	PKC
**cg07836663**	**PRKCZ**	1p36.33	8.27326E-13	7.15637E-07	66.22	73.87	−7.65	0.76	0.66	0.87	PKC

These include CpG sites with Target ID, ABC, SLC, and PKC Genes ID, chromosome location, *p*-value, FDR *p*-value, and percentage of methylation change. The bold values indicate markers both hyper and hypo methylated.

### Protein-protein interaction network

**FIGURE 2:**
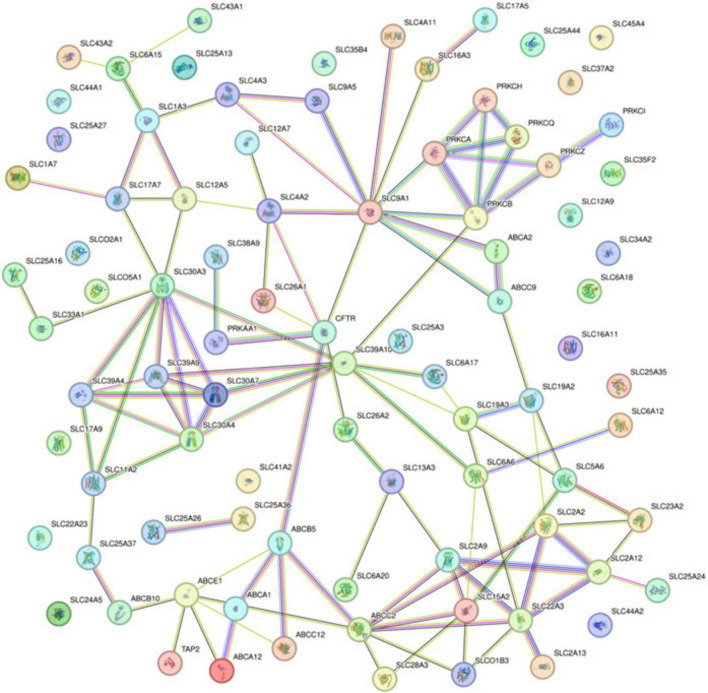
Protein-Protein Interaction Network from STRING Analysis for Differentially Methylated Genes. Interactions are depicted as lines connecting nodes, which represent individual proteins. Line colors correspond to the type of evidence supporting the interaction: red for gene fusion; green for the neighborhood; blue for co-occurrence; purple for experimental; yellow for text mining; light blue for database evidence; and black for co-expression.

The Protein-Protein Interaction (PPI) network analysis, utilizing the STRING database, delineated the complex interaction landscape among the 87 differentially methylated genes (Figure 2). The generated network comprised 87 nodes, each representing an individual gene product, connected by 110 edges that signified the putative protein-protein interactions. With an average node degree of 2.53, the network demonstrated a modest level of connectivity, where, on average, a protein was associated with approximately two and a half other proteins. The network’s average local clustering coefficient stood at 0.402, indicating a moderate propensity for proteins to form clusters, suggesting the presence of functional groupings within the network. The number of edges observed in the network significantly exceeded what would be expected in a random set of proteins, with an actual edge count of 110 compared to an expected count of 10. This substantial difference, validated by a PPI enrichment *p*-value < 1.0e–16, implies that the interactions are statistically significant and likely to be biologically relevant, rather than occurring by mere chance.

Specifically, noteworthy interactions were observed between kinase and transporter proteins. *PRKCB* showed connections with *SLC39A10* and *SLC9A1*, while *PRKCA* was linked to *SLC9A1*. Additionally, *PRKAA1* exhibited interactions with *CFTR* (*ABCC7*) and *SLC38A9*. These kinase and transporter protein interactions underscore potential regulatory points critical to the molecular mechanisms of NOWS.

### GO and KEGG pathway enrichment

**FIGURE 3:**
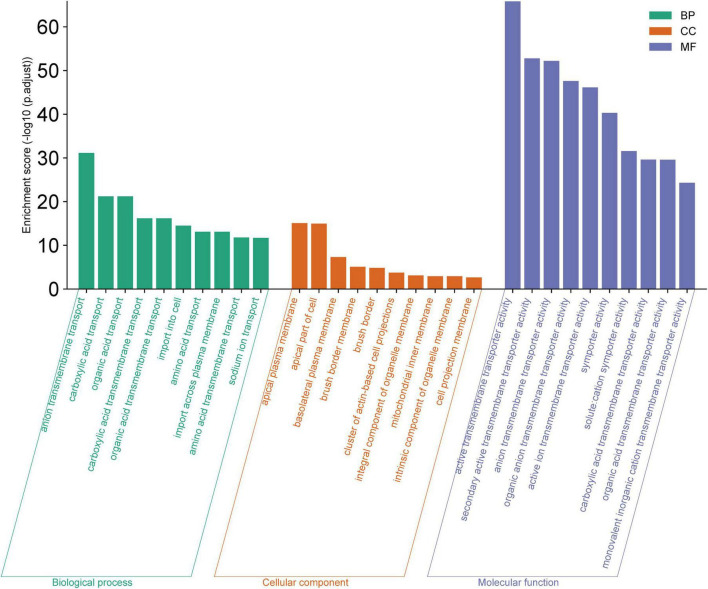
Gene Ontology (GO) Analysis for Biological Process (BP), Cellular Component (CC), and Molecular Function (MF). The bar chart displays the enrichment scores (−log10 (p.adjust) for the top ten GO terms within each category. Elevated enrichment scores indicate a higher level of significance, suggesting that these GO terms are notably overrepresented in the dataset, and may be integral to the underlying mechanisms of the biological system being investigated.

The GO analysis highlighted significant enrichments in biological processes (BP) primarily related to various substance transport activities, particularly anion and carboxylic acid transport. The cellular components (CC) most represented were those associated with the cell membrane, including the apical plasma membrane and basolateral plasma membrane. Molecular functions (MF) predominantly involved active transport activities, with a focus on transmembrane transporter activities (Figure 3). The GO analysis enrichment scores and related details are present in Supplementary Tables 5–7.

**FIGURE 4:**
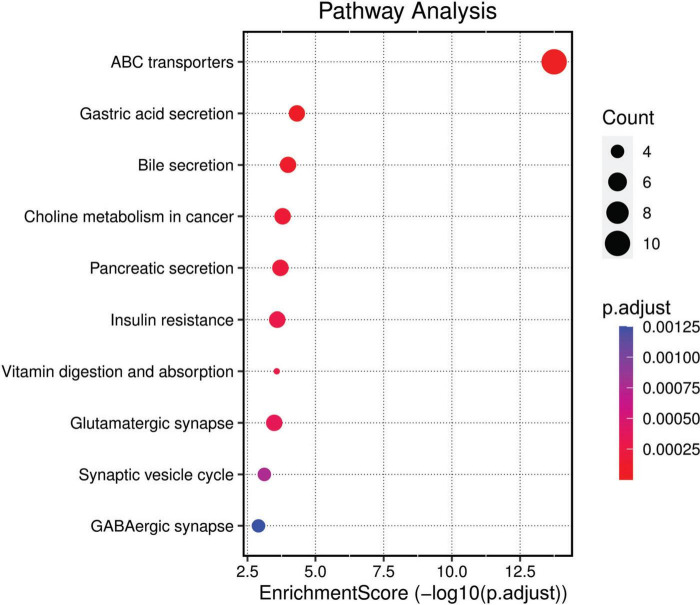
KEGG Pathway Analysis. This bubble plot visualizes the enriched pathways among the differently methylated genes based on the Kyoto Encyclopaedia of Genes and Genomes (KEGG) database. Pathways are listed on the y-axis and are ordered by the enrichment score (−log10(p.adjust)), plotted on the x-axis. The size of each bubble corresponds to the gene count within the pathway, and the color gradient represents the adjusted *p*-value, with darker hues indicating higher significance.

KEGG pathway analysis revealed that the differently methylated genes were significantly associated with 13 different pathways including ABC transporters, Gastric acid secretion, Bile secretion, Choline metabolism in cancer, Pancreatic secretion, Insulin resistance, Vitamin digestion, and absorption, Glutamatergic synapse, Synaptic vesicle cycle, GABAergic synapse, Salivary secretion, Inflammatory mediator regulation of TRP channels and Mineral absorption. Among them, the ‘ABC transporters’ pathway was prominent, suggesting a potential role in the transport of a wide range of substrates across extra- and intracellular membranes. Other notable pathways included those related to digestive system functions, metabolism, and synaptic neurotransmission, which could be linked to the biological underpinnings of NOWS. The visual representation and distribution of the enrichment scores across these pathways highlight the key biological processes potentially disrupted in NOWS (Figure 4). Complete details regarding the KEGG pathway analysis are provided in Supplementary Table 8.

### Evaluation of heatmaps

**FIGURE 5:**
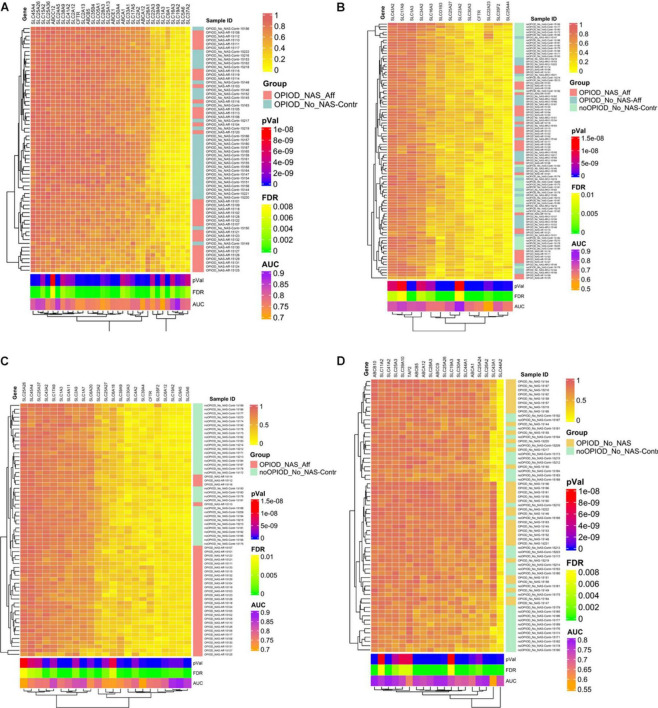
Heatmap displaying normalized beta values representing the top differentially methylated CpG sites in subjects exposed to prenatal opioids, along with CpG sites associated with transporters and protein kinase C (PKCs). Beta values for CpG sites with an unadjusted *p* < 0.005 and |Δβ| ≥ 0.05 are shown. Each row contains individual samples (affected and controls), and the columns pertain to the beta value for a CpG site color-coded from 0-1 (see color key, top right). The analysis was done in four combinations. Hierarchical sorting was performed by the CpG-associated genes site (columns), and the dendrogram (left) indicates similarities in methylation trends across all CpG sites. **(A)** Analysis I was conducted to distinguish NOWS from prenatal opioid exposure without NOWS symptoms. Heatmap comparing +Opioids/+NOWS and +Opioids/-NOWS reveals significant differences in methylation status among SLC and ABC drug transporter genes, alongside PKCs family members linked to NOWS. **(B)** Analysis II was conducted to distinguish prenatal opioid abuse from normal controls (OUD detection). Heatmap analyzing (+Opioids/+NOWS), (+Opioids/-NOWS), versus (-Opioids/-NOWS, control). **(C)** Analysis III was conducted to distinguish NOWS from unexposed controls. Heatmap displaying a comparison of (+Opioids/+NOWS) versus (-Opioids/-NOWS, control). **(D)** Analysis IV was conducted to distinguish opioid-induced epigenetic changes. Heatmap displaying analysis of (+Opioids/-NOWS), versus (-Opioids/-NOWS, control).

The heatmap, driven by CpG methylation markers associated with transporters and PKCs, clearly delineates distinct clusters: one for NOWS and the other for the control group. This compelling evidence underscores the reliability of these methylation markers in distinguishing between NOWS-affected patients and unaffected individuals, as demonstrated across all four distinct analyses presented in Figures 5A–D. In essence, our findings corroborate the accuracy and efficacy of these methylation markers in accurately discriminating between the two study groups.

## Discussion

Treating NOWS effectively remains challenging due to the paradoxical risks associated with unknown molecular and long-term developmental consequences. While short-term symptoms are observable soon after birth, uncertainties about long-term effects underscore the need for extensive studies. The detailed dysregulation of significant methylation changes in SLC, ABC transporters, and PKC genes associated with NOWS is described below.

*ABCA1* is involved in cholesterol and lipid transport (Oram, 2003). Variations in *ABCA1* can cause Tangier disease, characterized by impaired lipid efflux from macrophages leading to early atherosclerosis and low HDL levels (Peters et al., 2022).

*ABCA12* transports lipids like glucosylceramides across cell membranes to create the skin’s protective outer layer, the stratum corneum, shielding against environmental damage and preventing water loss (Akiyama, 2011). Variations in *ABCA12* can cause autosomal recessive congenital ichthyosis, marked by abnormal skin scaling and dryness (Hotz et al., 2023). Dry skin is common among opioid users or those addicted to opioids.

*ABCA2* plays a role in regulating cholesterol homeostasis in the brain, facilitating lipid transport, and impacting drug resistance in cancer cells, notably tumor stem cells. Variations in this gene are associated with early-onset Alzheimer’s disease (AD) (Mack et al., 2008). ABCA2 also exhibits resistance to compounds like estradiol and mitoxantrone (Mack et al., 2007).

*ABCB10* plays a crucial role in transporting porphyrins, necessary for heme biosynthesis and mitochondrial function (Yamamoto et al., 2014), its variations can impact heme metabolism and mitochondrial function, potentially influencing cellular responses to oxidative stress or drug-induced toxicity (Martinez et al., 2020).

*ABCB5* plays a key role in multidrug resistance in cancer cells, exporting chemotherapy drugs and reducing their effectiveness (Muriithi et al., 2020).

*ABCC12*, a multidrug resistance protein (MRP), removes drugs, toxins, and metabolites from cells, playing a crucial role in drug resistance. Variations in *ABCC12* are linked to breast cancer, liver hepatocellular carcinoma (Meng et al., 2022), bile duct paucity, cholestatic liver disease (Pham et al., 2021), and may also affect spermatid development and sperm function (Ono et al., 2007).

*ABCC2* and *ABCC9* genes, encoding multidrug resistance-associated proteins (MRPs), play crucial roles in drug resistance by regulating drug efflux, particularly in chemotherapy and antiretroviral therapy (Sodani et al., 2012). *ABCC2* expels glucuronidated metabolites and bilirubin, impacting liver function and inflammation (Choudhuri and Klaassen, 2006). *ABCC2* gene variants contribute to susceptibility to nonalcoholic fatty liver disease (NAFLD) (Sookoian et al., 2009), which is prevalent in cases of NAFLD with cirrhosis, high BMI, and psychiatric disorders involving opioid use (Moon et al., 2021). *ABCC9* expressed in the cardiac, smooth muscle, and brain, influences drug responses in cardiovascular and neurological contexts, thereby impacting overall drug efficacy and resistance (Nelson et al., 2015) and often causing sudden cardiac death (Subbotina et al., 2019). Infants born to opioid-addicted mothers have experienced sudden and unexpected deaths (Pierson et al., 1972).

*ABCC7* (*CFTR*) gene encodes a chloride channel critical for regulating ion transport and maintaining salt and water balance in tissues like the lungs, pancreas, and intestines (Lukasiak and Zajac, 2021). Dysfunction of this protein is linked to cystic fibrosis and can contribute to multidrug resistance in specific cell types. Variations in *CFTR* disrupt glucose homeostasis, leading to drug resistance through complex metabolic and cellular mechanisms (Ntimbane et al., 2009).

*ABCE1* is a multifunctional protein involved in viral replication and cellular antiviral responses. In HIV pathogenesis, it aids in viral core disassembly and HIV-1 capsid formation (Ramnani et al., 2021). *ABCE1* is also associated with chemotherapy and broader drug resistance mechanisms (Tian et al., 2012).

*ABCB3* is crucial for antigen presentation and contributes to multidrug resistance (Fan et al., 2023). Variations in *ABCB3* affect susceptibility to viral infections, autoimmune diseases, autoinflammatory diseases, and certain cancers (Mantel et al., 2022).

### The SLC transporters

#### Telomere-associated genes

We identified nine dysregulated genes involved in telomere maintenance.^1^ (*ABCC12, ABCC2, ABCC9, CFTR, SLC25A36, SLC39A10, SLC6A12, PRKCB, PRKCQ*) Accelerated telomere shortening in maternal cells may result in increased cellular aging, reduced regenerative capacity, and heightened susceptibility to age-related pathologies (Bar and Blasco, 2016). Compromised telomere maintenance during fetal development can lead to abnormalities and a higher risk of congenital defects due to its role in genomic stability (Tardat and Dejardin, 2018). Maternal opioid use increases the risk of telomere shortening (Rahimi Mehdi Abad et al., 2021).

#### Glucose metabolism-related genes

Maternal opioid exposure can modify glucose metabolism and insulin sensitivity, potentially influencing gene expression and the function of glucose transport pathways critical for drug resistance (Toorie et al., 2022). This disruption can affect fetal nutrient supply and development, potentially predisposing offspring to future metabolic issues (Toorie et al., 2021). We identified six key genes (*SLC16A3, SLC19A2, SLC25A13, SLC2A2, SLC37A2*, and *PRKAA1*) crucial for glucose metabolism.

#### Circadian rhythm dysregulation

Circadian rhythms are 24-h cycles regulating physiological processes like sleep, hormone release, and metabolism. Ultradian rhythms, occurring within a day, include REM and non-REM sleep, feeding, and hormone release. Sleep insufficiency and circadian disruptions correlate with obesity, cardiovascular disease, and cognitive impairment, though mechanisms are unclear (Chaput et al., 2023). We identified nine genes associated with circadian rhythm: *ABCA1, SLC12A9, SLC22A23, SLC25A37, SLC43A1, SLC45A4, SLC4A2, SLC6A12*, and *SLC6A6*. These genes are crucial for lipid transport (*ABCA1*) (Phillips, 2018), electrolyte balance (*SLC12A9*) (Levin-Konigsberg et al., 2023), and neurotransmitter transport (*SLC6A12, SLC6A6*) (Pramod et al., 2013). *PRKCA*, regulates circadian and ultradian rhythms, particularly sleep and hormonal cycles (Moller-Levet et al., 2013; Hu et al., 2023). Genes associated with ultradian rhythms include *SLC23A2, SLC24A5, SLC25A37*, and *SLC6A6*, influencing vitamin C transport (*SLC23A2*) (Kobayashi et al., 2024) and calcium transport (*SLC24A5*) (Quillen and Shriver, 2008).

#### Suicide-associated genes

The risk of suicide death is significantly higher in individuals with opioid use disorders (Rizk et al., 2021). We identified dysregulations in four PKC genes—*PRKCA, PRKCB, PRKCH*, and *PRKCI*—known for their roles in mood regulation and stress response, crucial for neurodevelopment and linked to psychiatric disorders (Choi et al., 2011; Coon et al., 2020; Sokolowski and Wasserman, 2020; Pandey et al., 2021). Additionally, the SLC genes *SLC19A2, SLC1A3, SLC4A2*, and *SLC4A3* are implicated in suicide through their roles in neural function and neurotransmitter regulation*: SLC19A2* encodes a thiamine transporter essential for brain metabolism; thiamine deficiencies can lead to mood dysregulation and suicidal behavior (Lutz et al., 2017). *SLC1A3* encodes a glutamate transporter, and its dysregulation is linked to depression and anxiety, significant risk factors for suicide (Murphy et al., 2011). *SLC4A2* regulates brain pH balance and is linked to mood disorders, observed in individuals with suicidal tendencies (Coon et al., 2020; Mirza et al., 2024). *SLC4A3* encodes a bicarbonate transporter; its dysfunction can disturb the brain’s acid-base balance, possibly contributing to neurological and psychiatric disorders, noted for significant variability in individuals who have died by suicide (Punzi et al., 2022).

#### Metal ion transporters

Zinc, cobalt, manganese, and magnesium transporters support infants’ cellular balance, neuronal development, and drug resistance. Zinc is crucial for growth, brain development, and neuronal function (Li et al., 2022). Dysfunctional zinc transporters that hinder zinc uptake in the brain can disrupt neuronal signaling and synaptic plasticity, potentially impairing cognitive development and increasing the risk of neurological disorders later in life (Wang et al., 2023). The six SLC transporters (*SLC30A3, SLC30A4, SLC30A7, SLC39A10, SLC39A4, SLC39A9*) are crucial for zinc balance, affecting immune function, growth, and neuronal development. Other metal-related genes like *SLC12A5* (cobalt carrier), *SLC11A2* (manganese transporter), and *SLC41A2* (magnesium transporter) significantly affect the immediate and long-term health of infants exposed to opioids in utero. Cobalt is vital for vitamin B12 production, hematopoiesis, immune responses, and antibacterial activities. Disruptions in cobalt levels can cause anemia, neurological issues, impaired hypoxia response, and drug resistance (Ma et al., 2022). Increased *SLC11A2* activity can lead to excessive manganese uptake, contributing to neurodegenerative disorders such as Parkinson’s disease, impacting immune function, and potentially causing severe toxicity from manganese accumulation (Chen et al., 2015). Magnesium deficiency linked to dysregulated *SLC41A2* can lead to neurological symptoms like seizures and muscle spasms, affecting neural function and overall health (Al Alawi et al., 2018).

#### Oxidative stress-related genes

We identified *PRKAA1* (PKC gene) and five SLC genes (*SLC17A5, SLC1A3, SLC23A2, SLC25A27, SLC6A6*) linked to oxidative stress. *PRKAA1*, a regulator of cellular energy homeostasis activated under stress conditions, enhances antioxidant defenses and cellular repair in opioid exposure. However, chronic activation may alter metabolic states, impacting drug metabolism and resistance. *SLC17A5* disruption by oxidative stress affects lysosomal function, impairing drug metabolism (Ruivo et al., 2009). *SLC1A3* dysfunction due to oxidative stress causes neuronal damage and affects drug response (Ayka and Sehirli, 2020). *SLC23A2*, crucial for vitamin C uptake, combats oxidative stress; impaired function reduces antioxidant capacity (Teafatiller et al., 2021). *SLC25A27* (*UCP4*), involved in mitochondrial ROS reduction, influences drug metabolism under chronic oxidative stress (Zhang et al., 2024). *SLC6A6* alteration in taurine transport affects cellular resilience, potentially influencing drug resistance mechanisms (Baliou et al., 2021).

#### Opioid use is known to increase impulsivity

Impulsivity is common in opioid addiction cases (Tolomeo et al., 2021). We identified three genes linked to impulsivity—*SLCO5A1, PRKCA*, and *PRKCH*—in infants born to mothers with opioid use disorders.

*SLCO5A1* regulates drug transport across cell membranes, influencing neurodevelopmental processes linked to impulsivity (Roshandel et al., 2023). *PRKCA* regulates signal transduction, influencing neurotransmitter signaling and synaptic plasticity, thereby increasing susceptibility to impulsive behaviors while *PRKCH* is critical for neuronal signaling and brain development; its dysregulation may disrupt brain development, potentially increasing impulsivity (Khadka et al., 2014). Animal studies similarly indicate that prenatal opioid exposure results in long-term cognitive deficits and impulsivity (Alaee et al., 2021).

#### Protein Kinase C family (PKC)

PKC family members play crucial roles in cell signaling and serve as therapeutic targets for various conditions, including diabetes, cancer, cardiovascular issues, dermatological conditions, psychiatric disorders, neurological diseases, and immune-mediated ailments (Mochly-Rosen et al., 2012). Dysregulation of the seven PKCs—*PRKCA, PRKCB, PRKCH, PRKCI, PRKCQ*, and PRKAA1—is linked to specific diseases and disorders. Both *PRKCA* and *PRKCH* are associated with impulsivity, as previously explained. Variations in both *PRKCB* and *PRKCQ* play significant roles in certain cancers and are considered excellent predictive biomarkers for these diseases (Abdelatty et al., 2021). Chronic opioid therapy increases the risk of cancer in noncancer patients with chronic pain (Oh and Song, 2020).

#### Pathways

We found significant epigenetic changes in multiple genes across 13 pathways, outlining important dysregulated pathways linked to NOWS and OUD.

ABC Transporters: ABC transporters are pivotal in drug resistance as they actively expel drugs from cells, diminishing their therapeutic efficacy. Moreover, these transporters can influence the pharmacokinetics of drugs of abuse, affecting their distribution and elimination from the body. In the placenta, ABC transporters significantly reduce fetal exposure to drugs and other foreign substances, potentially influencing therapy for NOWS (Gottesman and Ambudkar, 2001).

Pancreatic Secretion: Altered pancreatic secretion can affect the absorption of certain drugs, potentially impacting their efficacy and bioavailability (Olesen et al., 2013). Pancreatic dysfunction may occur in individuals with substance abuse disorders, affecting drug metabolism and pancreatic health (Jones et al., 2015). Neonatal exposure to drugs affecting pancreatic function can lead to digestive disturbances and contribute to NOWS.

Bile Secretion: Bile secretion can affect the enterohepatic circulation of drugs and their metabolites, impacting drug levels and efficacy. Disruption of bile secretion pathways may contribute to drug-induced liver injury and drug resistance (Li and Apte, 2015). Neonatal exposure to drugs affecting bile secretion can lead to cholestasis and NOWS.

Insulin Resistance: Insulin resistance is associated with metabolic disorders often seen in substance abuse, potentially affecting drug metabolism and response. Substance abuse can contribute to insulin resistance, exacerbating metabolic complications and drug resistance. Neonatal exposure to substances affecting insulin sensitivity may influence fetal growth and contribute to NOWS.

Glutamatergic Synapse. Disruption of the glutamatergic system is linked to addiction and drug tolerance, altering responses to addictive substances (Alasmari et al., 2022). Targeting glutamatergic synapses offers promise in treating drug addiction and reducing drug resistance. Early exposure to drugs impacting glutamatergic neurotransmission in neonates may affect brain development and contribute to NOWS.

Synaptic Vesicle Cycle: Disruption of the synaptic vesicle cycle can alter neurotransmitter release, affecting the rewarding effects of addictive substances (Sulzer, 2011). Drugs of abuse can modulate the synaptic vesicle cycle, leading to long-term changes in synaptic function and drug tolerance (Wang W. et al., 2019). Prenatal exposure to substances affecting the synaptic vesicle cycle may influence neuronal development and contribute to NOWS.

Gastric Acid Secretion: Alterations in gastric acid secretion can affect the absorption and efficacy of certain medications used in addiction treatment. Gastric acid secretion may influence the absorption of drugs of abuse, affecting their onset and duration of action (Bushra et al., 2011). Neonates exposed to maternal substance abuse may experience gastric disturbances contributing to NOWS.

Inflammatory Mediator Regulation of Transient Receptor Potential (TRP) Channels. TRP channels play a role in pain perception and neuroinflammation associated with substance abuse and addiction (Yang et al., 2023). Inflammatory mediators can modulate TRP channel activity, influencing the development of drug tolerance and withdrawal symptoms. Neonatal exposure to drugs affecting TRP channels and inflammatory mediators may impact sensory processing and contribute to NOWS.

## Conclusion

In summary, our study enhances our understanding of the epigenetic basis of NOWS, emphasizing the roles of PKC and drug transporters. Methylation changes in these genes may serve as NOWS biomarkers, opening new research and clinical avenues. These findings aim to mitigate maternal OUD and relapse, enhance care for opioid-exposed infants, and support their families. Future steps include investigating the functional impacts of these changes and developing effective therapies for NOWS and OUD management. Exploring epigenetic influences on drug metabolism could improve global patient care by enhancing drug safety and efficacy.

## Data availability statement

The original contributions presented in the study are included in the article/Supplementary material, further inquiries can be directed to the corresponding author.

## Ethics statement

The studies involving humans were approved by the Institutional Review Board of Beaumont Health System, Royal Oak, MI, USA (HIC#: 2019-086). The studies were conducted in accordance with the local legislation and institutional requirements. The human samples used in this study were acquired from primarily isolated as part of your previous study for which ethical approval was obtained. Written informed consent for participation was not required from the participants or the participants’ legal guardians/next of kin in accordance with the national legislation and institutional requirements.

## Author contributions

UR: Conceptualization, Data curation, Formal analysis, Funding acquisition, Investigation, Methodology, Project administration, Resources, Software, Supervision, Validation, Visualization, Writing−original draft, Writing−review and editing. RuR: Data curation, Formal analysis, Investigation, Methodology, Validation, Visualization, Writing−original draft, Writing−review and editing. LU: Data curation, Formal analysis, Investigation, Methodology, Software, Validation, Visualization, Writing−original draft, Writing−review and editing. SM: Formal analysis, Investigation, Methodology, Project administration, Validation, Visualization, Writing−original draft, Writing−review and editing. JP: Data curation, Formal analysis, Methodology, Software, Validation, Visualization, Writing−review and editing. RMR: Conceptualization, Data curation, Investigation, Methodology, Software, Validation, Visualization, Writing−review and editing, Writing−original draft. RB-S: Conceptualization, Data curation, Investigation, Supervision, Validation, Visualization, Writing−review and editing. SS: Data curation, Formal analysis, Investigation, Resources, Software, Supervision, Validation, Visualization, Writing−original draft, Writing−review and editing.

## Footnotes

1
https://malone2.bioquant.uni-heidelberg.de/fmi/webd/TelNet
