# Supplementary material for: Prenatal opioid exposure significantly impacts placental protein kinase C (PKC) and drug transporters, leading to drug resistance and neonatal opioid withdrawal syndrome
Source: Front Neurosci. 2024 Aug 19;18:1442915. doi: 10.3389/fnins.2024.1442915 (PMC11376091; doi:10.3389/fnins.2024.1442915)
Supplement: Supplementary file 1 [file Data_Sheet_1.docx]

**Supplementary Table-S1.** The comparison between +Opioids/+NOWS and +Opioids/-NOWS shows distinct differences in the methylation status of SLC and ABC drug transporter genes, as well as PKC family members associated with NOWS.

| **Target ID** | **Gene** | **CHR** | **p-Val** | **FDR p-Val** | **% Methylation** | | | **CI** | | | **Gene  Details** |
| --- | --- | --- | --- | --- | --- | --- | --- | --- | --- | --- | --- |
|  |  |  |  |  | **Cases** | **Control** | **Change** | **AUC** | **lower** | **upper** |  |
| cg10434274 | ABCA1 | 9 | 4.14763E-09 | 0.003587703 | 60.83 | 68.83 | -8.00 | 0.75 | 0.63 | 0.87 | ABC |
| cg20618167 | ABCA12 | 2 | 6.45958E-11 | 5.58753E-05 | 50.17 | 59.71 | -9.54 | 0.71 | 0.58 | 0.84 | ABC |
| cg21045171 | ABCB10 | 1 | 2.66472E-08 | 0.023049813 | 78.01 | 83.60 | -5.59 | 0.75 | 0.64 | 0.87 | ABC |
| cg08888968 | ABCB5 | 7 | 2.90402E-16 | 2.51198E-10 | 68.08 | 77.76 | -9.69 | 0.76 | 0.64 | 0.88 | ABC |
| cg21723907 | ABCC12 | 16 | 8.67446E-09 | 0.007503411 | 74.56 | 80.83 | -6.28 | 0.78 | 0.67 | 0.89 | ABC |
| cg01614760 | ABCC2 | 10 | 2.00132E-08 | 0.017311439 | 43.41 | 35.80 | 7.60 | 0.74 | 0.62 | 0.86 | ABC |
| cg22533025 | ABCC7 /CFTR | 7 | 1.97867E-11 | 1.71155E-05 | 72.00 | 79.58 | -7.59 | 0.73 | 0.61 | 0.85 | ABC |
| cg26690672 | ABCE1 | 4 | 2.09819E-12 | 1.81494E-06 | 63.82 | 72.88 | -9.06 | 0.76 | 0.64 | 0.88 | ABC |
| cg02131891 | SLC15A2 | 3 | 2.47989E-09 | 0.002145104 | 78.88 | 84.68 | -5.80 | 0.83 | 0.73 | 0.94 | SLC |
| cg15639045 | SLC16A11 | 17 | 3.49989E-08 | 0.03027406 | 55.79 | 63.69 | -7.90 | 0.76 | 0.65 | 0.88 | SLC |
| cg03956820 | SLC16A3 | 17 | 4.63951E-09 | 0.004013176 | 17.56 | 12.01 | 5.55 | 0.84 | 0.74 | 0.94 | SLC |
| cg13460167 | SLC17A5 | 6 | 9.83035E-10 | 0.000850325 | 56.82 | 65.43 | -8.61 | 0.78 | 0.67 | 0.90 | SLC |
| cg00959118 | SLC19A2 | 1 | 7.31371E-10 | 0.000632636 | 17.10 | 11.39 | 5.71 | 0.89 | 0.80 | 0.97 | SLC |
| cg06960901 | SLC1A3 | 5 | 4.06686E-09 | 0.003517831 | 27.60 | 20.80 | 6.80 | 0.70 | 0.58 | 0.83 | SLC |
| cg01739295 | SLC22A3 | 6 | 3.72641E-09 | 0.003223343 | 34.96 | 27.49 | 7.47 | 0.73 | 0.60 | 0.85 | SLC |
| cg13223777 | SLC24A5 | 15 | 1.26703E-14 | 1.09598E-08 | 72.94 | 81.33 | -8.39 | 0.85 | 0.76 | 0.95 | SLC |
| cg12030923 | SLC25A13 | 7 | 3.85551E-10 | 0.000333502 | 64.94 | 72.99 | -8.04 | 0.73 | 0.61 | 0.85 | SLC |
| cg14710071 | SLC25A16 | 10 | 4.75308E-08 | 0.041114139 | 80.54 | 85.65 | -5.11 | 0.78 | 0.66 | 0.89 | SLC |
| cg04773602 | SLC25A24 | 1 | 2.06183E-08 | 0.017834839 | 18.05 | 12.64 | 5.42 | 0.74 | 0.62 | 0.86 | SLC |
| cg00298230 | SLC25A26 | 3 | 4.97067E-11 | 4.29963E-05 | 79.20 | 85.45 | -6.25 | 0.85 | 0.75 | 0.94 | SLC |
| cg14775474 | SLC25A35 | 17 | 1.44284E-08 | 0.01248054 | 11.75 | 7.23 | 4.52 | 0.85 | 0.75 | 0.94 | SLC |
| cg18270394 | SLC25A36 | 3 | 1.46857E-11 | 1.27031E-05 | 77.30 | 84.04 | -6.74 | 0.75 | 0.63 | 0.87 | SLC |
| cg09153458 | SLC26A1 | 4 | 4.46293E-13 | 3.86043E-07 | 55.56 | 45.90 | 9.66 | 0.77 | 0.65 | 0.88 | SLC |
| cg26303603 | SLC26A2 | 5 | 1.71203E-08 | 0.014809033 | 47.20 | 55.60 | -8.40 | 0.75 | 0.63 | 0.87 | SLC |
| cg12588047 | SLC28A3 | 9 | 8.78757E-10 | 0.000760125 | 67.46 | 75.07 | -7.60 | 0.71 | 0.59 | 0.84 | SLC |
| cg00309135 | SLC2A12 | 6 | 1.74595E-09 | 0.001510246 | 71.49 | 78.46 | -6.97 | 0.77 | 0.66 | 0.89 | SLC |
| cg07645864 | SLC2A13 | 12 | 4.7902E-11 | 4.14352E-05 | 71.55 | 79.07 | -7.53 | 0.73 | 0.61 | 0.85 | SLC |
| cg19132526 | SLC2A2 | 3 | 8.64142E-12 | 7.47483E-06 | 51.94 | 61.80 | -9.86 | 0.78 | 0.67 | 0.89 | SLC |
| cg16989032 | SLC30A4 | 15 | 3.04023E-12 | 2.6298E-06 | 61.32 | 70.61 | -9.29 | 0.76 | 0.64 | 0.88 | SLC |
| cg02530515 | SLC30A7 | 1 | 1.18431E-08 | 0.010244289 | 69.42 | 76.32 | -6.90 | 0.76 | 0.65 | 0.88 | SLC |
| cg11178666 | SLC33A1 | 3 | 2.61279E-09 | 0.002260067 | 56.99 | 65.38 | -8.40 | 0.76 | 0.64 | 0.87 | SLC |
| cg16584327 | SLC35B4 | 7 | 3.26095E-11 | 2.82072E-05 | 69.14 | 77.07 | -7.93 | 0.75 | 0.63 | 0.87 | SLC |
| cg14469376 | SLC37A2 | 11 | 4.6003E-09 | 0.003979261 | 9.77 | 5.46 | 4.31 | 0.78 | 0.67 | 0.89 | SLC |
| cg02765475 | SLC38A9 | 5 | 4.08508E-09 | 0.003533594 | 75.04 | 81.37 | -6.33 | 0.75 | 0.64 | 0.87 | SLC |
| cg17926678 | SLC39A9 | 14 | 1.62919E-38 | 1.40925E-32 | 34.99 | 24.49 | 10.50 | 0.77 | 0.65 | 0.88 | SLC |
| cg25287207 | SLC41A2 | 12 | 2.1923E-09 | 0.001896343 | 71.73 | 78.63 | -6.90 | 0.80 | 0.69 | 0.91 | SLC |
| cg02426178 | SLC44A2 | 19 | 2.01983E-08 | 0.01747157 | 80.20 | 85.49 | -5.29 | 0.70 | 0.57 | 0.83 | SLC |
| cg08203794 | SLC45A4 | 8 | 2.12648E-11 | 1.8394E-05 | 83.88 | 77.62 | 6.26 | 0.81 | 0.70 | 0.92 | SLC |
| cg20078681 | SLC4A3 | 2 | 1.86189E-08 | 0.016105309 | 84.71 | 89.24 | -4.53 | 0.85 | 0.76 | 0.95 | SLC |
| cg13250541 | SLC5A6 | 2 | 2.1925E-09 | 0.001896509 | 13.97 | 8.87 | 5.10 | 0.79 | 0.68 | 0.90 | SLC |
| cg22081654 | SLC6A12 | 12 | 4.59955E-08 | 0.039786143 | 21.05 | 15.36 | 5.68 | 0.75 | 0.63 | 0.87 | SLC |
| cg04394707 | SLC6A15 | 12 | 2.35189E-08 | 0.020343858 | 60.54 | 68.20 | -7.66 | 0.72 | 0.60 | 0.85 | SLC |
| cg16321159 | SLC6A17 | 1 | 3.57318E-08 | 0.030907997 | 81.60 | 86.58 | -4.98 | 0.76 | 0.65 | 0.88 | SLC |
| cg06617455 | SLC6A6 | 3 | 1.60204E-08 | 0.013857662 | 51.42 | 59.71 | -8.29 | 0.76 | 0.65 | 0.88 | SLC |
| cg22367705 | SLC9A1 | 1 | 1.4567E-14 | 1.26004E-08 | 18.10 | 11.05 | 7.05 | 0.78 | 0.66 | 0.89 | SLC |
| cg12878682 | SLCO5A1 | 8 | 2.47908E-12 | 2.14441E-06 | 68.47 | 76.89 | -8.42 | 0.74 | 0.62 | 0.86 | SLC |
| cg08532220 | PRKCI | 3 | 2.61116E-10 | 0.000225865 | 57.36 | 66.20 | -8.84 | 0.74 | 0.62 | 0.86 | PKC |
| cg00105154 | PRKCQ | 10 | 9.19432E-14 | 7.95308E-08 | 21.46 | 14.05 | 7.42 | 0.75 | 0.63 | 0.87 | PKC |

**Supplementary Table-S2.** Analysis of (+Opioids/+NOWS), + (+Opioids/-NOWS), versus (-Opioids/-NOWS, control). CpG targets with significant methylation differences in SLC and ABC drug transporter genes, as well as PKC family members associated with NOWS.

| **Target ID** | **Gene** |  | **p-value** | **FDR p-Val** | **% Methylation** | | |  | **CI** | | **Gene**  **Details** |
| --- | --- | --- | --- | --- | --- | --- | --- | --- | --- | --- | --- |
|  |  | **CHR** |  |  | **Cases** | **Control** | **Change** | **AUC** | **lower** | **upper** |  |
| cg02820283 | ABCA2 | 9 | 2.81873E-08 | 0.02438205 | 72.63 | 78.14 | -5.51 | 0.71 | 0.60 | 0.83 | ABC |
| cg26635219 | ABCC7 /CFTR | 7 | 4.77178E-13 | 4.12759E-07 | 20.30 | 14.19 | 6.11 | 0.64 | 0.52 | 0.76 | ABC |
| cg08296680 | SLC16A3 | 17 | 3.54873E-09 | 0.00306965 | 44.17 | 51.88 | -7.72 | 0.67 | 0.55 | 0.79 | SLC |
| cg12285003 | SLC17A9 | 20 | 1.08447E-08 | 0.00938070 | 73.03 | 78.63 | -5.60 | 0.78 | 0.67 | 0.88 | SLC |
| cg03989758 | SLC1A3 | 5 | 5.7521E-11 | 4.97556E-05 | 70.66 | 77.23 | -6.58 | 0.73 | 0.62 | 0.85 | SLC |
| cg11713788 | SLC22A23 | 6 | 1.14405E-09 | 0.00098960 | 15.53 | 21.56 | -6.03 | 0.55 | 0.43 | 0.68 | SLC |
| cg17978727 | SLC23A2 | 20 | 1.12949E-08 | 0.00977005 | 34.28 | 27.91 | 6.37 | 0.84 | 0.75 | 0.93 | SLC |
| cg25217269 | SLC25A27 | 6 | 1.95076E-11 | 1.68741E-05 | 29.90 | 38.17 | -8.27 | 0.73 | 0.62 | 0.84 | SLC |
| cg16405055 | SLC25A44 | 1 | 1.02268E-10 | 8.84615E-05 | 7.03 | 11.86 | -4.83 | 0.65 | 0.53 | 0.77 | SLC |
| cg08789022 | SLC30A3 | 2 | 8.59211E-14 | 7.43217E-08 | 21.71 | 15.24 | 6.47 | 0.68 | 0.56 | 0.80 | SLC |
| cg02272859 | SLC34A2 | 4 | 7.09098E-09 | 0.00613370 | 59.64 | 66.60 | -6.96 | 0.75 | 0.64 | 0.86 | SLC |
| cg26453171 | SLC35F2 | 11 | 2.49321E-11 | 2.15662E-05 | 14.53 | 20.97 | -6.44 | 0.72 | 0.61 | 0.83 | SLC |
| cg19272348 | SLC43A2 | 17 | 7.2856E-09 | 0.00630204 | 75.29 | 80.66 | -5.37 | 0.73 | 0.62 | 0.85 | SLC |
| cg19392998 | SLC44A2 | 19 | 2.90447E-08 | 0.02512370 | 6.64 | 10.70 | -4.06 | 0.65 | 0.53 | 0.77 | SLC |
| cg17156227 | SLC4A11 | 20 | 4.5614E-08 | 0.03945608 | 68.39 | 74.28 | -5.90 | 0.69 | 0.57 | 0.81 | SLC |
| cg25268697 | SLCO1B3 | 12 | 6.83856E-12 | 5.91536E-06 | 35.62 | 44.35 | -8.74 | 0.66 | 0.54 | 0.78 | SLC |
| cg22822824 | SLCO2A1 | 3 | 3.31677E-08 | 0.02869003 | 6.18 | 10.14 | -3.96 | 0.61 | 0.48 | 0.73 | SLC |
| cg13127598 | PRKCB | 16 | 1.76442E-08 | 0.01526223 | 18.23 | 24.17 | -5.94 | 0.57 | 0.45 | 0.70 | PKC |
| cg18417061 | PRKCH | 14 | 6.21097E-10 | 0.00053725 | 76.05 | 81.62 | -5.57 | 0.81 | 0.71 | 0.91 | PKC |
| cg07836663 | PRKCZ | 1 | 8.27326E-13 | 7.15637E-07 | 66.22 | 73.87 | -7.65 | 0.76 | 0.66 | 0.87 | PKC |
| cg26294217 | PRKAA1 | 5 | 2.58104E-08 | 0.02232597 | 6.11 | 10.08 | -3.98 | 0.74 | 0.63 | 0.85 | PKC |

**Supplementary Table-S3.** Comparison of (+Opioids/+NOWS) versus (-Opioids/-NOWS, control), highlighting CpG targets showing significant methylation differences in SLC and ABC drug transporter genes, alongside PKC family members linked to NOWS.

| **Target ID** | **Gene** | **CHR** | **p-Val** | **FDR p-Val** | **% Methylation** | | | **AUC** | **CI** | | **Gene**  **Details** |
| --- | --- | --- | --- | --- | --- | --- | --- | --- | --- | --- | --- |
|  |  |  |  |  | **Cases** | **Control** | **Change** |  | **lower** | **upper** |  |
| cg26635219 | ABCC7 /CFTR | 7 | 9.66969E-39 | 8.36428E-33 | 24.59 | 14.19 | 10.40 | 0.76 | 0.64 | 0.88 | ABC |
| cg10439765 | SLC12A5 | 20 | 2.61284E-08 | 0.022601057 | 15.53 | 21.81 | -6.27 | 0.70 | 0.57 | 0.82 | SLC |
| cg18756954 | SLC12A7 | 5 | 2.03828E-08 | 0.017631088 | 72.18 | 78.71 | -6.53 | 0.74 | 0.62 | 0.86 | SLC |
| cg15062310 | SLC12A9 | 7 | 5.60196E-08 | 0.048456948 | 14.05 | 9.28 | 4.77 | 0.78 | 0.66 | 0.89 | SLC |
| cg09192862 | SLC13A3 | 20 | 2.0586E-08 | 0.017806874 | 79.71 | 85.13 | -5.42 | 0.84 | 0.74 | 0.94 | SLC |
| cg02624701 | SLC17A7 | 19 | 1.26807E-08 | 0.010968811 | 30.60 | 23.62 | 6.98 | 0.71 | 0.58 | 0.84 | SLC |
| cg12285003 | SLC17A9 | 20 | 3.10992E-09 | 0.002690084 | 71.71 | 78.63 | -6.91 | 0.83 | 0.73 | 0.93 | SLC |
| cg00959118 | SLC19A2 | 1 | 1.03149E-09 | 0.000892237 | 17.13 | 11.40 | 5.73 | 0.87 | 0.78 | 0.96 | SLC |
| cg03989758 | SLC1A3 | 5 | 1.52389E-11 | 1.31816E-05 | 69.13 | 77.23 | -8.10 | 0.76 | 0.65 | 0.88 | SLC |
| cg05414613 | SLC1A7 | 1 | 1.97336E-11 | 1.70696E-05 | 62.49 | 71.39 | -8.90 | 0.82 | 0.72 | 0.93 | SLC |
| cg13282929 | SLC23A2 | 20 | 1.05376E-11 | 9.11502E-06 | 38.36 | 48.43 | -10.07 | 0.78 | 0.66 | 0.89 | SLC |
| cg13223777 | SLC24A5 | 15 | 5.70281E-08 | 0.049329284 | 74.03 | 80.13 | -6.11 | 0.75 | 0.63 | 0.87 | SLC |
| cg06555256 | SLC25A26 | 3 | 1.0893E-08 | 0.00942241 | 82.55 | 87.58 | -5.03 | 0.70 | 0.57 | 0.83 | SLC |
| cg25217269 | SLC25A27 | 6 | 2.82907E-09 | 0.002447147 | 29.73 | 38.17 | -8.44 | 0.71 | 0.59 | 0.84 | SLC |
| cg03084648 | SLC25A37 | 8 | 5.46944E-09 | 0.00473107 | 77.37 | 83.36 | -5.99 | 0.77 | 0.65 | 0.88 | SLC |
| cg20566657 | SLC2A9 | 4 | 3.53496E-09 | 0.003057739 | 64.98 | 72.69 | -7.71 | 0.82 | 0.72 | 0.92 | SLC |
| cg08789022 | SLC30A3 | 2 | 2.09826E-38 | 1.815E-32 | 24.84 | 15.24 | 9.60 | 0.75 | 0.63 | 0.87 | SLC |
| cg02272859 | SLC34A2 | 4 | 1.42711E-08 | 0.012344479 | 58.59 | 66.60 | -8.01 | 0.78 | 0.67 | 0.89 | SLC |
| cg26453171 | SLC35F2 | 11 | 4.69575E-12 | 4.06182E-06 | 13.57 | 20.97 | -7.40 | 0.77 | 0.65 | 0.88 | SLC |
| cg14228592 | SLC39A4 | 8 | 8.63426E-11 | 7.46864E-05 | 21.89 | 15.16 | 6.74 | 0.70 | 0.57 | 0.83 | SLC |
| cg17926678 | SLC39A9 | 14 | 1.63699E-10 | 0.000141599 | 35.33 | 27.23 | 8.11 | 0.73 | 0.60 | 0.85 | SLC |
| cg19272348 | SLC43A2 | 17 | 2.95112E-10 | 0.000255272 | 73.67 | 80.66 | -6.99 | 0.76 | 0.65 | 0.88 | SLC |
| cg08203794 | SLC45A4 | 8 | 6.79724E-09 | 0.005879614 | 85.62 | 80.41 | 5.20 | 0.75 | 0.63 | 0.87 | SLC |
| cg17156227 | SLC4A11 | 20 | 8.58965E-12 | 7.43005E-06 | 65.62 | 74.28 | -8.66 | 0.79 | 0.67 | 0.90 | SLC |
| cg09988421 | SLC4A2 | 7 | 1.9444E-09 | 0.001681904 | 26.37 | 19.51 | 6.86 | 0.78 | 0.67 | 0.90 | SLC |
| cg13250541 | SLC5A6 | 2 | 2.56509E-14 | 2.2188E-08 | 13.99 | 7.73 | 6.26 | 0.85 | 0.75 | 0.95 | SLC |
| cg26758670 | SLC6A12 | 12 | 4.31442E-10 | 0.000373197 | 18.40 | 12.37 | 6.03 | 0.79 | 0.68 | 0.90 | SLC |
| cg17277001 | SLC6A18 | 5 | 8.83477E-09 | 0.007642076 | 28.79 | 36.89 | -8.10 | 0.71 | 0.58 | 0.84 | SLC |
| cg26339753 | SLC6A20 | 3 | 1.3871E-09 | 0.001199845 | 58.65 | 67.14 | -8.49 | 0.73 | 0.61 | 0.86 | SLC |
| cg13476831 | SLC9A5 | 16 | 1.00782E-09 | 0.000871768 | 15.73 | 10.21 | 5.52 | 0.88 | 0.79 | 0.96 | SLC |
| cg07836663 | PRKCZ | 1 | 1.23891E-10 | 0.000107166 | 65.64 | 73.87 | -8.23 | 0.79 | 0.68 | 0.90 | PKC |

**Supplementary Table-****S4.** Analysis of (+Opioids/-NOWS), versus (-Opioids/-NOWS, control) with details of significantly differentially methylated CpGs in SLC and ABC drug transporter genes, and PKC family members associated with NOWS.

| **Target ID** | **Gene** | **CHR** | **p-Val** | **FDR p-Val** | **% Methylation** | | | **AUC** | **CI** | | **Gene**  **Details** |
| --- | --- | --- | --- | --- | --- | --- | --- | --- | --- | --- | --- |
|  |  |  |  |  | **Cases** | **Control** | **Change** |  | **lower** | **upper** |  |
| cg14299235 | ABCA1 | 9 | 5.50932E-10 | 0.000476556 | 67.75 | 59.84 | 7.91 | 0.68 | 0.55 | 0.81 | ABC |
| cg00972111 | ABCA12 | 2 | 4.72015E-14 | 4.08293E-08 | 78.32 | 70.24 | 8.08 | 0.73 | 0.61 | 0.85 | ABC |
| cg21045171 | ABCB10 | 1 | 2.51239E-11 | 2.17322E-05 | 85.30 | 79.30 | 6.00 | 0.79 | 0.68 | 0.90 | ABC |
| cg08888968 | ABCB5 | 7 | 9.25029E-39 | 8.0015E-33 | 80.18 | 70.50 | 9.68 | 0.75 | 0.63 | 0.87 | ABC |
| cg01909678 | ABCC9 | 12 | 3.14029E-14 | 2.71635E-08 | 76.39 | 67.95 | 8.44 | 0.78 | 0.67 | 0.89 | ABC |
| cg00720839 | ABCB3 /TAP2 | 6 | 6.28153E-09 | 0.005433526 | 80.38 | 74.32 | 6.06 | 0.64 | 0.50 | 0.78 | ABC |
| cg22826226 | SLC11A2 | 12 | 7.94208E-09 | 0.006869897 | 80.63 | 74.65 | 5.98 | 0.76 | 0.65 | 0.88 | SLC |
| cg24865549 | SLC17A5 | 6 | 2.09993E-08 | 0.018164393 | 85.53 | 80.50 | 5.04 | 0.72 | 0.60 | 0.85 | SLC |
| cg09726804 | SLC19A3 | 2 | 8.28861E-09 | 0.007169646 | 73.34 | 66.44 | 6.90 | 0.73 | 0.61 | 0.86 | SLC |
| cg17949403 | SLC22A23 | 6 | 1.62173E-08 | 0.014027981 | 63.91 | 56.38 | 7.53 | 0.70 | 0.57 | 0.83 | SLC |
| cg17978727 | SLC23A2 | 20 | 3.2701E-08 | 0.028286374 | 35.05 | 27.91 | 7.14 | 0.86 | 0.77 | 0.95 | SLC |
| cg23045610 | SLC25A24 | 1 | 1.71658E-09 | 0.001484844 | 64.20 | 56.25 | 7.95 | 0.77 | 0.65 | 0.88 | SLC |
| cg10343071 | SLC25A26 | 3 | 5.46218E-11 | 4.72479E-05 | 73.11 | 65.32 | 7.79 | 0.78 | 0.66 | 0.89 | SLC |
| cg25217269 | SLC25A27 | 6 | 3.57635E-08 | 0.030935455 | 30.30 | 38.17 | -7.86 | 0.74 | 0.62 | 0.86 | SLC |
| cg27155504 | SLC25A3 | 12 | 3.46705E-09 | 0.002999001 | 80.86 | 74.78 | 6.08 | 0.77 | 0.65 | 0.88 | SLC |
| cg26303603 | SLC26A2 | 5 | 2.80194E-11 | 2.42368E-05 | 59.10 | 50.17 | 8.93 | 0.78 | 0.67 | 0.89 | SLC |
| cg12588047 | SLC28A3 | 9 | 2.14091E-14 | 1.85189E-08 | 77.39 | 69.01 | 8.38 | 0.75 | 0.63 | 0.87 | SLC |
| cg07645864 | SLC2A13 | 12 | 4.80512E-08 | 0.041564282 | 81.05 | 75.43 | 5.62 | 0.73 | 0.61 | 0.86 | SLC |
| cg16989032 | SLC30A4 | 15 | 1.74464E-11 | 1.50912E-05 | 73.48 | 65.55 | 7.93 | 0.70 | 0.57 | 0.83 | SLC |
| cg00003999 | SLC39A10 | 2 | 6.45175E-09 | 0.005580765 | 79.37 | 73.18 | 6.20 | 0.74 | 0.61 | 0.86 | SLC |
| cg25287207 | SLC41A2 | 12 | 6.23886E-11 | 5.39661E-05 | 80.33 | 73.56 | 6.77 | 0.79 | 0.68 | 0.90 | SLC |
| cg07291744 | SLC43A1 | 11 | 2.60106E-39 | 2.24991E-33 | 56.96 | 45.99 | 10.97 | 0.58 | 0.44 | 0.72 | SLC |
| cg22521553 | SLC44A1 | 9 | 1.22738E-12 | 1.06169E-06 | 73.51 | 65.18 | 8.33 | 0.71 | 0.58 | 0.83 | SLC |
| cg19392998 | SLC44A2 | 19 | 7.21249E-11 | 6.2388E-05 | 5.57 | 10.70 | -5.13 | 0.73 | 0.60 | 0.85 | SLC |
| cg11899080 | PRKCA | 17 | 5.88738E-39 | 5.09258E-33 | 65.29 | 55.15 | 10.15 | 0.82 | 0.71 | 0.92 | PKC |
| cg00105154 | PRKCQ | 10 | 5.72985E-13 | 4.95632E-07 | 14.86 | 22.82 | -7.97 | 0.70 | 0.57 | 0.83 | PKC |
| cg16269144 | PRKCZ | 1 | 3.26129E-10 | 0.000282101 | 80.02 | 73.44 | 6.58 | 0.77 | 0.65 | 0.89 | PKC |
| cg07836663 | PRKCZ | 1 | 3.10214E-08 | 0.026833472 | 66.79 | 73.87 | -7.08 | 0.74 | 0.62 | 0.86 | PKC |
| cg05878107 | PRKCI | 3 | 7.36142E-39 | 6.36763E-33 | 72.02 | 62.10 | 9.92 | 0.77 | 0.66 | 0.89 | PKC |
| cg26294217 | PRKAA1 | 5 | 6.80901E-09 | 0.005889795 | 5.54 | 10.08 | -4.54 | 0.76 | 0.65 | 0.88 | PKC |
