# Supplementary material for: Prenatal opioid exposure significantly impacts placental protein kinase C (PKC) and drug transporters, leading to drug resistance and neonatal opioid withdrawal syndrome
Source: Front Neurosci. 2024 Aug 19;18:1442915. doi: 10.3389/fnins.2024.1442915 (PMC11376091; doi:10.3389/fnins.2024.1442915)
Supplement: Supplementary file 2 [file Data_Sheet_2.docx]

**Supplementary Table-S5.** The Gene Ontology Analysis reveals significant enrichments in biological processes (BP) among differentially methylated SLC and ABC drug transporter genes in NOWS.

| **ID** | **Description** | **p-value** | **FDR** | **Gene ID** |
| --- | --- | --- | --- | --- |
| GO:0098656 | Anion transmembrane transport | 4.83058E-35 | 7.04781E-32 | CFTR/SLC12A5/SLC12A7/SLC12A9/SLC13A3/SLC16A11/SLC16A3/SLC17A7/SLC1A3/SLC1A7/SLC23A2/SLC24A5/SLC25A13/SLC25A3/SLC26A1/SLC26A2/SLC35B4/SLC37A2/SLC38A9/SLC43A1/SLC43A2/SLC4A11/SLC4A2/SLC4A3/SLC5A6/SLC6A12/SLC6A15/SLC6A18/SLC6A20/SLC6A6/SLC9A1/SLC9A5 |
| GO:0046942 | Carboxylic acid transport | 9.88891E-25 | 5.99784E-22 | ABCC2/SLC13A3/SLC16A11/SLC16A3/SLC17A5/SLC17A7/SLC19A2/SLC1A3/SLC1A7/SLC23A2/SLC25A13/SLC25A44/SLC26A1/SLC26A2/SLC38A9/SLC43A1/SLC43A2/SLC5A6/SLC6A12/SLC6A15/SLC6A17/SLC6A18/SLC6A20/SLC6A6/SLCO1B3/SLCO2A1 |
| GO:0015849 | Organic acid transport | 1.23328E-24 | 5.99784E-22 | ABCC2/SLC13A3/SLC16A11/SLC16A3/SLC17A5/SLC17A7/SLC19A2/SLC1A3/SLC1A7/SLC23A2/SLC25A13/SLC25A44/SLC26A1/SLC26A2/SLC38A9/SLC43A1/SLC43A2/SLC5A6/SLC6A12/SLC6A15/SLC6A17/SLC6A18/SLC6A20/SLC6A6/SLCO1B3/SLCO2A1 |
| GO:1905039 | Carboxylic acid transmembrane transport | 1.99728E-19 | 6.54461E-17 | SLC13A3/SLC16A11/SLC16A3/SLC17A7/SLC1A3/SLC1A7/SLC23A2/SLC25A13/SLC38A9/SLC43A1/SLC43A2/SLC5A6/SLC6A12/SLC6A15/SLC6A18/SLC6A20/SLC6A6 |
| GO:1903825 | Organic acid transmembrane transport | 2.24284E-19 | 6.54461E-17 | SLC13A3/SLC16A11/SLC16A3/SLC17A7/SLC1A3/SLC1A7/SLC23A2/SLC25A13/SLC38A9/SLC43A1/SLC43A2/SLC5A6/SLC6A12/SLC6A15/SLC6A18/SLC6A20/SLC6A6 |
| GO:0098657 | import into cell | 1.32673E-17 | 3.22616E-15 | ABCC9/SLC11A2/SLC12A5/SLC12A7/SLC12A9/SLC15A2/SLC1A3/SLC1A7/SLC22A3/SLC39A10/SLC39A4/SLC43A1/SLC43A2/SLC5A6/SLC6A20/SLC6A6/SLC9A1/SLC9A5 |
| GO:0006865 | amino acid transport | 3.83057E-16 | 7.69656E-14 | SLC17A5/SLC17A7/SLC1A3/SLC1A7/SLC25A13/SLC25A44/SLC38A9/SLC43A1/SLC43A2/SLC6A12/SLC6A15/SLC6A17/SLC6A18/SLC6A20/SLC6A6 |
| GO:0098739 | import across plasma membrane | 4.22018E-16 | 7.69656E-14 | ABCC9/SLC12A5/SLC12A7/SLC12A9/SLC15A2/SLC1A3/SLC39A10/SLC39A4/SLC43A1/SLC43A2/SLC5A6/SLC6A20/SLC6A6/SLC9A1/SLC9A5 |
| GO:0003333 | amino acid transmembrane transport | 9.80783E-15 | 1.58996E-12 | SLC17A7/SLC1A3/SLC1A7/SLC25A13/SLC38A9/SLC43A1/SLC43A2/SLC6A12/SLC6A15/SLC6A18/SLC6A20/SLC6A6 |
| GO:0006814 | sodium ion transport | 1.33299E-14 | 1.94483E-12 | SLC13A3/SLC17A7/SLC23A2/SLC24A5/SLC28A3/SLC34A2/SLC4A11/SLC5A6/SLC6A12/SLC6A15/SLC6A17/SLC6A18/SLC6A20/SLC6A6/SLC9A1/SLC9A5 |
| GO:0015803 | branched-chain amino acid transport | 2.47822E-14 | 3.28702E-12 | SLC25A44/SLC38A9/SLC43A1/SLC43A2/SLC6A15/SLC6A17/SLC6A20 |
| GO:0015698 | inorganic anion transport | 4.12252E-14 | 5.0123E-12 | CFTR/SLC12A5/SLC12A7/SLC12A9/SLC17A7/SLC1A3/SLC25A3/SLC26A1/SLC26A2/SLC34A2/SLC37A2/SLC4A11/SLC4A2/SLC4A3 |
| GO:0072348 | sulfur compound transport | 9.79084E-13 | 1.09883E-10 | SLC13A3/SLC19A2/SLC19A3/SLC25A26/SLC26A1/SLC26A2/SLC33A1/SLC5A6/SLC6A6 |
| GO:0035725 | sodium ion transmembrane transport | 1.07847E-12 | 1.12392E-10 | SLC17A7/SLC24A5/SLC28A3/SLC34A2/SLC4A11/SLC6A12/SLC6A15/SLC6A17/SLC6A18/SLC6A20/SLC6A6/SLC9A1/SLC9A5 |
| GO:0098661 | inorganic anion transmembrane transport | 7.75993E-12 | 7.54783E-10 | CFTR/SLC12A5/SLC12A7/SLC12A9/SLC17A7/SLC1A3/SLC25A3/SLC26A1/SLC26A2/SLC37A2/SLC4A11 |
| GO:1901264 | carbohydrate derivative transport | 1.49737E-11 | 1.36542E-09 | SLC17A5/SLC25A13/SLC25A16/SLC25A24/SLC25A36/SLC25A44/SLC28A3/SLC33A1/SLC35B4/SLC37A2 |
| GO:0015807 | L-amino acid transport | 4.23512E-11 | 3.63473E-09 | SLC17A7/SLC1A3/SLC1A7/SLC25A13/SLC38A9/SLC43A1/SLC43A2/SLC6A17/SLC6A20 |
| GO:0008643 | carbohydrate transport | 8.7424E-11 | 7.0862E-09 | PRKCB/PRKCI/SLC17A5/SLC23A2/SLC25A27/SLC2A12/SLC2A2/SLC2A9/SLC35B4/SLC37A2/SLC45A4 |
| GO:1902600 | proton transmembrane transport | 1.52707E-10 | 1.17263E-08 | SLC11A2/SLC15A2/SLC17A5/SLC17A7/SLC25A27/SLC25A3/SLC2A9/SLC33A1/SLC4A11/SLC9A1/SLC9A5 |
| GO:1902475 | L-alpha-amino acid transmembrane transport | 3.69973E-10 | 2.69895E-08 | SLC17A7/SLC1A3/SLC1A7/SLC25A13/SLC38A9/SLC43A1/SLC43A2/SLC6A20 |
| GO:0006835 | dicarboxylic acid transport | 6.26087E-10 | 4.34981E-08 | ABCC2/SLC13A3/SLC17A7/SLC19A2/SLC1A3/SLC1A7/SLC25A13/SLC26A1/SLC26A2 |
| GO:0015804 | neutral amino acid transport | 8.99849E-10 | 5.96763E-08 | SLC38A9/SLC43A1/SLC43A2/SLC6A15/SLC6A17/SLC6A20/SLC6A6 |
| GO:0006829 | zinc ion transport | 2.06033E-09 | 1.30697E-07 | SLC30A3/SLC30A4/SLC30A7/SLC39A10/SLC39A4/SLC39A9 |
| GO:0051503 | adenine nucleotide transport | 6.19365E-09 | 3.76522E-07 | SLC25A13/SLC25A16/SLC25A24/SLC25A36/SLC25A44/SLC33A1 |
| GO:0000041 | transition metal ion transport | 7.27273E-09 | 4.23251E-07 | ABCC2/SLC11A2/SLC25A37/SLC30A3/SLC30A4/SLC30A7/SLC39A10/SLC39A4/SLC39A9 |
| GO:0015868 | purine ribonucleotide transport | 7.54251E-09 | 4.23251E-07 | SLC25A13/SLC25A16/SLC25A24/SLC25A36/SLC25A44/SLC33A1 |
| GO:0015865 | purine nucleotide transport | 9.12549E-09 | 4.93115E-07 | SLC25A13/SLC25A16/SLC25A24/SLC25A36/SLC25A44/SLC33A1 |
| GO:0015748 | organophosphate ester transport | 1.08497E-08 | 5.65348E-07 | ABCA1/ABCA12/SLC25A13/SLC25A16/SLC25A24/SLC25A36/SLC25A44/SLC33A1/SLC37A2 |
| GO:0055081 | anion homeostasis | 1.28083E-08 | 6.33806E-07 | ABCA1/ABCC2/PRKAA1/SLC12A5/SLC12A7/SLC12A9/SLC34A2 |
| GO:0098659 | inorganic cation import across plasma membrane | 1.39012E-08 | 6.33806E-07 | ABCC9/SLC12A5/SLC12A7/SLC12A9/SLC39A10/SLC39A4/SLC9A1/SLC9A5 |
| GO:0099587 | inorganic ion import across plasma membrane | 1.39012E-08 | 6.33806E-07 | ABCC9/SLC12A5/SLC12A7/SLC12A9/SLC39A10/SLC39A4/SLC9A1/SLC9A5 |
| GO:1902476 | chloride transmembrane transport | 1.39012E-08 | 6.33806E-07 | CFTR/SLC12A5/SLC12A7/SLC12A9/SLC17A7/SLC1A3/SLC26A1/SLC26A2 |
| GO:0006862 | nucleotide transport | 1.84612E-08 | 8.16208E-07 | SLC25A13/SLC25A16/SLC25A24/SLC25A36/SLC25A44/SLC33A1 |
| GO:0015701 | bicarbonate transport | 4.00331E-08 | 1.68141E-06 | CFTR/SLC26A1/SLC26A2/SLC4A11/SLC4A2/SLC4A3 |
| GO:0006821 | chloride transport | 4.03353E-08 | 1.68141E-06 | CFTR/SLC12A5/SLC12A7/SLC12A9/SLC17A7/SLC1A3/SLC26A1/SLC26A2 |
| GO:0043090 | amino acid import | 5.30978E-08 | 2.15193E-06 | SLC17A7/SLC1A3/SLC43A1/SLC43A2/SLC6A20/SLC6A6 |
| GO:0034219 | carbohydrate transmembrane transport | 6.51265E-08 | 2.5681E-06 | PRKCB/PRKCI/SLC17A5/SLC23A2/SLC25A27/SLC2A12/SLC2A2/SLC2A9 |
| GO:0006836 | neurotransmitter transport | 9.02386E-08 | 3.46469E-06 | PRKCB/SLC17A7/SLC1A3/SLC1A7/SLC22A3/SLC6A12/SLC6A15/SLC6A17/SLC6A18/SLC6A6 |
| GO:0055083 | monovalent inorganic anion homeostasis | 1.06742E-07 | 3.99324E-06 | ABCC2/SLC12A5/SLC12A7/SLC12A9/SLC34A2 |
| GO:0015867 | ATP transport | 1.58342E-07 | 5.77552E-06 | SLC25A13/SLC25A16/SLC25A24/SLC25A36/SLC25A44 |
| GO:0072531 | pyrimidine-containing compound transmembrane transport | 1.93731E-07 | 6.89398E-06 | SLC19A2/SLC19A3/SLC25A36/SLC28A3 |
| GO:0055064 | chloride ion homeostasis | 3.89049E-07 | 1.35148E-05 | ABCC2/SLC12A5/SLC12A7/SLC12A9 |
| GO:0055067 | monovalent inorganic cation homeostasis | 6.48478E-07 | 2.2003E-05 | CFTR/SLC12A5/SLC12A7/SLC12A9/SLC4A2/SLC4A3/SLC9A1/SLC9A5 |
| GO:0006882 | cellular zinc ion homeostasis | 6.80022E-07 | 2.25489E-05 | SLC30A3/SLC30A4/SLC30A7/SLC39A10/SLC39A4 |
| GO:0055069 | zinc ion homeostasis | 8.91806E-07 | 2.89143E-05 | SLC30A3/SLC30A4/SLC30A7/SLC39A10/SLC39A4 |
| GO:0035461 | vitamin transmembrane transport | 9.15404E-07 | 2.90342E-05 | SLC19A2/SLC19A3/SLC23A2/SLC5A6 |
| GO:0015749 | monosaccharide transmembrane transport | 9.96146E-07 | 3.09229E-05 | PRKCB/PRKCI/SLC23A2/SLC25A27/SLC2A12/SLC2A2/SLC2A9 |
| GO:0051180 | vitamin transport | 1.15249E-06 | 3.43159E-05 | SLC19A2/SLC19A3/SLC23A2/SLC2A2/SLC5A6 |
| GO:0089718 | amino acid import across plasma membrane | 1.15249E-06 | 3.43159E-05 | SLC1A3/SLC43A1/SLC43A2/SLC6A20/SLC6A6 |
| GO:0015718 | monocarboxylic acid transport | 1.2821E-06 | 3.74116E-05 | ABCC2/SLC16A11/SLC16A3/SLC5A6/SLC6A12/SLC6A6/SLCO1B3/SLCO2A1 |
| GO:0072511 | divalent inorganic cation transport | 1.70413E-06 | 4.87516E-05 | ABCC2/PRKCB/SLC11A2/SLC24A5/SLC30A3/SLC30A4/SLC30A7/SLC39A10/SLC39A4/SLC39A9/SLC41A2/SLC9A1 |
| GO:0015931 | nucleobase-containing compound transport | 2.93867E-06 | 8.24522E-05 | ABCE1/SLC25A13/SLC25A16/SLC25A24/SLC25A36/SLC25A44/SLC28A3/SLC33A1/SLC35B4 |
| GO:0055076 | transition metal ion homeostasis | 3.19321E-06 | 8.79037E-05 | SLC11A2/SLC25A37/SLC30A3/SLC30A4/SLC30A7/SLC39A10/SLC39A4 |
| GO:0015813 | L-glutamate transmembrane transport | 3.3355E-06 | 8.84816E-05 | SLC17A7/SLC1A3/SLC1A7/SLC25A13 |
| GO:0071577 | zinc ion transmembrane transport | 3.3355E-06 | 8.84816E-05 | SLC30A3/SLC30A4/SLC39A10/SLC39A4 |
| GO:0015850 | organic hydroxy compound transport | 3.6474E-06 | 9.50279E-05 | ABCA1/ABCA12/ABCA2/ABCC2/CFTR/SLC16A3/SLC22A3/SLC2A13/SLCO1B3 |
| GO:0070633 | transepithelial transport | 5.57292E-06 | 0.000142647 | ABCC2/CFTR/SLC1A3/SLC23A2 |
| GO:0006817 | phosphate ion transport | 6.51943E-06 | 0.000161218 | SLC17A7/SLC25A3/SLC34A2/SLC37A2 |
| GO:0072337 | modified amino acid transport | 6.51943E-06 | 0.000161218 | SLC13A3/SLC19A2/SLC5A6/SLC6A20 |
| GO:0071805 | potassium ion transmembrane transport | 6.84382E-06 | 0.000166419 | ABCC9/SLC12A5/SLC12A7/SLC12A9/SLC1A3/SLC24A5/SLC9A1/SLC9A5 |
| GO:0070838 | divalent metal ion transport | 9.46652E-06 | 0.000226421 | PRKCB/SLC11A2/SLC24A5/SLC30A3/SLC30A4/SLC30A7/SLC39A10/SLC39A4/SLC39A9/SLC41A2/SLC9A1 |
| GO:0034350 | regulation of glial cell apoptotic process | 1.07242E-05 | 0.000248358 | PRKCA/PRKCH/PRKCI |
| GO:0050861 | positive regulation of B cell receptor signaling pathway | 1.07242E-05 | 0.000248358 | PRKCB/PRKCH/SLC39A10 |
| GO:1904659 | glucose transmembrane transport | 1.10251E-05 | 0.000251339 | PRKCB/PRKCI/SLC25A27/SLC2A12/SLC2A2/SLC2A9 |
| GO:0008645 | hexose transmembrane transport | 1.35301E-05 | 0.000303698 | PRKCB/PRKCI/SLC25A27/SLC2A12/SLC2A2/SLC2A9 |
| GO:0006813 | potassium ion transport | 1.46371E-05 | 0.00032065 | ABCC9/SLC12A5/SLC12A7/SLC12A9/SLC1A3/SLC24A5/SLC9A1/SLC9A5 |
| GO:0046627 | negative regulation of insulin receptor signaling pathway | 1.48872E-05 | 0.00032065 | PRKAA1/PRKCB/PRKCQ/PRKCZ |
| GO:0046916 | cellular transition metal ion homeostasis | 1.49446E-05 | 0.00032065 | SLC11A2/SLC30A3/SLC30A4/SLC30A7/SLC39A10/SLC39A4 |
| GO:0042908 | xenobiotic transport | 1.68137E-05 | 0.000355524 | ABCC2/SLC15A2/SLC22A3/SLC6A6 |
| GO:0015800 | acidic amino acid transport | 1.79871E-05 | 0.000374902 | SLC17A7/SLC1A3/SLC1A7/SLC25A13/SLC6A6 |
| GO:1900077 | negative regulation of cellular response to insulin stimulus | 1.89174E-05 | 0.000388738 | PRKAA1/PRKCB/PRKCQ/PRKCZ |
| GO:0015824 | proline transport | 1.95316E-05 | 0.000395786 | SLC6A15/SLC6A17/SLC6A20 |
| GO:0030002 | cellular anion homeostasis | 2.53074E-05 | 0.000498967 | ABCC2/SLC12A5/SLC34A2 |
| GO:0030320 | cellular monovalent inorganic anion homeostasis | 2.53074E-05 | 0.000498967 | ABCC2/SLC12A5/SLC34A2 |
| GO:0006839 | mitochondrial transport | 3.2947E-05 | 0.000640928 | ABCB10/PRKAA1/SLC25A13/SLC25A16/SLC25A24/SLC25A36/SLC25A37/SLC9A1 |
| GO:0034349 | glial cell apoptotic process | 3.9997E-05 | 0.000767837 | PRKCA/PRKCH/PRKCI |
| GO:0051453 | regulation of intracellular pH | 4.8213E-05 | 0.00090615 | CFTR/SLC4A2/SLC4A3/SLC9A1/SLC9A5 |
| GO:0043252 | sodium-independent organic anion transport | 4.9065E-05 | 0.00090615 | SLCO1B3/SLCO2A1/SLCO5A1 |
| GO:0051238 | sequestering of metal ion | 4.9065E-05 | 0.00090615 | SLC30A3/SLC30A4/SLC30A7 |
| GO:1990573 | potassium ion import across plasma membrane | 6.71993E-05 | 0.001225548 | ABCC9/SLC12A5/SLC12A7/SLC12A9 |
| GO:0030641 | regulation of cellular pH | 6.9857E-05 | 0.001258289 | CFTR/SLC4A2/SLC4A3/SLC9A1/SLC9A5 |
| GO:0015695 | organic cation transport | 7.29193E-05 | 0.00129743 | SLC22A3/SLC44A1/SLC44A2/SLC6A20 |
| GO:0006885 | regulation of pH | 0.000107892 | 0.001896557 | CFTR/SLC4A2/SLC4A3/SLC9A1/SLC9A5 |
| GO:0030004 | cellular monovalent inorganic cation homeostasis | 0.000173978 | 0.00302183 | CFTR/SLC4A2/SLC4A3/SLC9A1/SLC9A5 |
| GO:0140115 | export across plasma membrane | 0.000183531 | 0.003150258 | ABCA1/ABCC2/SLC15A2/SLC9A1 |
| GO:0015740 | C4-dicarboxylate transport | 0.000195628 | 0.003280702 | SLC13A3/SLC1A3/SLC25A13 |
| GO:0050857 | positive regulation of antigen receptor-mediated signaling pathway | 0.000195628 | 0.003280702 | PRKCB/PRKCH/SLC39A10 |
| GO:0046626 | regulation of insulin receptor signaling pathway | 0.000233856 | 0.003877226 | PRKAA1/PRKCB/PRKCQ/PRKCZ |
| GO:0050855 | regulation of B cell receptor signaling pathway | 0.000306728 | 0.004972403 | PRKCB/PRKCH/SLC39A10 |
| GO:0055075 | potassium ion homeostasis | 0.000306728 | 0.004972403 | SLC12A5/SLC12A7/SLC12A9 |
| GO:0006884 | cell volume homeostasis | 0.00033969 | 0.005446232 | SLC12A5/SLC12A7/SLC12A9 |
| GO:1900076 | regulation of cellular response to insulin stimulus | 0.000382293 | 0.006062674 | PRKAA1/PRKCB/PRKCQ/PRKCZ |
| GO:1903959 | regulation of anion transmembrane transport | 0.000412269 | 0.006467752 | CFTR/SLC43A1/SLC43A2 |
| GO:0018105 | peptidyl-serine phosphorylation | 0.000535603 | 0.00831325 | PRKAA1/PRKCA/PRKCB/PRKCH/PRKCI/PRKCQ/PRKCZ |
| GO:0032869 | cellular response to insulin stimulus | 0.000585335 | 0.008989515 | PRKAA1/PRKCB/PRKCI/PRKCQ/PRKCZ/SLC9A1 |
| GO:0006767 | water-soluble vitamin metabolic process | 0.000615219 | 0.009350049 | SLC19A2/SLC19A3/SLC23A2/SLC5A6 |
| GO:0016572 | histone phosphorylation | 0.000799907 | 0.011949071 | PRKAA1/PRKCA/PRKCB |
| GO:0051092 | positive regulation of NF-kappaB transcription factor activity | 0.000802611 | 0.011949071 | PRKCB/PRKCH/PRKCI/PRKCQ/PRKCZ |
| GO:0018209 | peptidyl-serine modification | 0.000816631 | 0.012034991 | PRKAA1/PRKCA/PRKCB/PRKCH/PRKCI/PRKCQ/PRKCZ |
| GO:0055088 | lipid homeostasis | 0.000897377 | 0.012910143 | ABCA1/ABCA12/ABCA2/PRKAA1/SLC25A27 |
| GO:0015851 | nucleobase transport | 0.00090256 | 0.012910143 | SLC23A2/SLC28A3 |
| GO:0015893 | drug transport | 0.00090256 | 0.012910143 | ABCC2/SLC22A3 |
| GO:0001504 | neurotransmitter uptake | 0.000923364 | 0.013042596 | SLC1A3/SLC1A7/SLC22A3 |
| GO:0042391 | regulation of membrane potential | 0.000929698 | 0.013042596 | ABCB5/CFTR/PRKCZ/SLC17A7/SLC25A27/SLC25A36/SLC4A3/SLC9A1 |
| GO:0006857 | oligopeptide transport | 0.001099862 | 0.01499719 | SLC13A3/SLC15A2 |
| GO:0019532 | oxalate transport | 0.001099862 | 0.01499719 | SLC26A1/SLC26A2 |
| GO:0070778 | L-aspartate transmembrane transport | 0.001099862 | 0.01499719 | SLC1A3/SLC25A13 |
| GO:0014047 | glutamate secretion | 0.001130155 | 0.015267559 | SLC17A7/SLC1A3/SLC1A7 |
| GO:0010874 | regulation of cholesterol efflux | 0.001205044 | 0.0161299 | ABCA1/ABCA12/ABCA2 |
| GO:0007077 | mitotic nuclear envelope disassembly | 0.001315929 | 0.016990619 | PRKCA/PRKCB |
| GO:0033700 | phospholipid efflux | 0.001315929 | 0.016990619 | ABCA1/ABCA12 |
| GO:0045117 | azole transmembrane transport | 0.001315929 | 0.016990619 | SLC19A2/SLC19A3 |
| GO:0060081 | membrane hyperpolarization | 0.001315929 | 0.016990619 | CFTR/PRKCZ |
| GO:0030301 | cholesterol transport | 0.001355607 | 0.017305571 | ABCA1/ABCA12/ABCA2/CFTR |
| GO:0009409 | response to cold | 0.001364044 | 0.017305571 | PRKAA1/SLC25A27/SLC9A1 |
| GO:0010038 | response to metal ion | 0.001410984 | 0.017746771 | ABCC2/PRKAA1/SLC11A2/SLC25A13/SLC25A24/SLC30A3/SLC30A4 |
| GO:0010958 | regulation of amino acid import across plasma membrane | 0.001550587 | 0.018696748 | SLC43A1/SLC43A2 |
| GO:0015697 | quaternary ammonium group transport | 0.001550587 | 0.018696748 | SLC22A3/SLC6A20 |
| GO:0015816 | glycine transport | 0.001550587 | 0.018696748 | SLC6A17/SLC6A20 |
| GO:0051956 | negative regulation of amino acid transport | 0.001550587 | 0.018696748 | SLC43A1/SLC43A2 |
| GO:1903789 | regulation of amino acid transmembrane transport | 0.001550587 | 0.018696748 | SLC43A1/SLC43A2 |
| GO:0035627 | ceramide transport | 0.001803668 | 0.021287292 | ABCA12/ABCA2 |
| GO:0051938 | L-glutamate import | 0.001803668 | 0.021287292 | SLC17A7/SLC1A3 |
| GO:0072503 | cellular divalent inorganic cation homeostasis | 0.001809201 | 0.021287292 | PRKCB/SLC24A5/SLC25A27/SLC30A3/SLC30A4/SLC30A7/SLC39A10/SLC39A4 |
| GO:0032868 | response to insulin | 0.001862337 | 0.021737194 | PRKAA1/PRKCB/PRKCI/PRKCQ/PRKCZ/SLC9A1 |
| GO:0032328 | alanine transport | 0.002075002 | 0.023535444 | SLC6A17/SLC6A6 |
| GO:0035435 | phosphate ion transmembrane transport | 0.002075002 | 0.023535444 | SLC25A3/SLC37A2 |
| GO:1903960 | negative regulation of anion transmembrane transport | 0.002075002 | 0.023535444 | SLC43A1/SLC43A2 |
| GO:0015918 | sterol transport | 0.002080927 | 0.023535444 | ABCA1/ABCA12/ABCA2/CFTR |
| GO:0006869 | lipid transport | 0.002113073 | 0.023715181 | ABCA1/ABCA12/ABCA2/ABCC2/CFTR/SLCO1B3/SLCO2A1 |
| GO:0015812 | gamma-aminobutyric acid transport | 0.002364421 | 0.025937522 | SLC6A12/SLC6A6 |
| GO:0030397 | membrane disassembly | 0.002364421 | 0.025937522 | PRKCA/PRKCB |
| GO:0051081 | nuclear envelope disassembly | 0.002364421 | 0.025937522 | PRKCA/PRKCB |
| GO:1904375 | regulation of protein localization to cell periphery | 0.00241989 | 0.026347901 | ABCA2/PRKCH/PRKCI/PRKCZ |
| GO:0015732 | prostaglandin transport | 0.00267176 | 0.02866248 | ABCC2/SLCO2A1 |
| GO:1902358 | sulfate transmembrane transport | 0.00267176 | 0.02866248 | SLC26A1/SLC26A2 |
| GO:0008272 | sulfate transport | 0.002996851 | 0.031684104 | SLC26A1/SLC26A2 |
| GO:0034755 | iron ion transmembrane transport | 0.002996851 | 0.031684104 | SLC11A2/SLC25A37 |
| GO:0006766 | vitamin metabolic process | 0.003038345 | 0.031891688 | SLC19A2/SLC19A3/SLC23A2/SLC5A6 |
| GO:0033344 | cholesterol efflux | 0.003118145 | 0.03249552 | ABCA1/ABCA12/ABCA2 |
| GO:0060252 | positive regulation of glial cell proliferation | 0.003339532 | 0.033835956 | PRKCH/PRKCI |
| GO:0090153 | regulation of sphingolipid biosynthetic process | 0.003339532 | 0.033835956 | ABCA2/PRKAA1 |
| GO:0098719 | sodium ion import across plasma membrane | 0.003339532 | 0.033835956 | SLC9A1/SLC9A5 |
| GO:1905038 | regulation of membrane lipid metabolic process | 0.003339532 | 0.033835956 | ABCA2/PRKAA1 |
| GO:0010875 | positive regulation of cholesterol efflux | 0.003699639 | 0.036971049 | ABCA1/ABCA12 |
| GO:1902004 | positive regulation of amyloid-beta formation | 0.003699639 | 0.036971049 | ABCA2/SLC2A13 |
| GO:0050854 | regulation of antigen receptor-mediated signaling pathway | 0.003857648 | 0.038287811 | PRKCB/PRKCH/SLC39A10 |
| GO:0010876 | lipid localization | 0.003949752 | 0.038937085 | ABCA1/ABCA12/ABCA2/ABCC2/CFTR/SLCO1B3/SLCO2A1 |
| GO:0071375 | cellular response to peptide hormone stimulus | 0.003983793 | 0.039009088 | PRKAA1/PRKCB/PRKCI/PRKCQ/PRKCZ/SLC9A1 |
| GO:0032374 | regulation of cholesterol transport | 0.00401724 | 0.039074355 | ABCA1/ABCA12/ABCA2 |
| GO:0090330 | regulation of platelet aggregation | 0.004077009 | 0.039393085 | PRKCA/PRKCQ |
| GO:0008286 | insulin receptor signaling pathway | 0.004156273 | 0.039867898 | PRKAA1/PRKCB/PRKCQ/PRKCZ |
| GO:0032371 | regulation of sterol transport | 0.004180801 | 0.039867898 | ABCA1/ABCA12/ABCA2 |
| GO:0009266 | response to temperature stimulus | 0.00425951 | 0.040354709 | ABCC2/PRKAA1/SLC12A5/SLC25A27/SLC9A1 |
| GO:0043434 | response to peptide hormone | 0.004303588 | 0.040509257 | ABCC2/PRKAA1/PRKCB/PRKCI/PRKCQ/PRKCZ/SLC9A1 |
| GO:0001505 | regulation of neurotransmitter levels | 0.004415855 | 0.041299571 | PRKCB/SLC17A7/SLC1A3/SLC1A7/SLC22A3 |
| GO:0032753 | positive regulation of interleukin-4 production | 0.005311093 | 0.048129721 | PRKCQ/PRKCZ |
| GO:0032891 | negative regulation of organic acid transport | 0.005311093 | 0.048129721 | SLC43A1/SLC43A2 |
| GO:0034035 | purine ribonucleoside bisphosphate metabolic process | 0.005311093 | 0.048129721 | SLC26A1/SLC26A2 |
| GO:0050427 | 3'-phosphoadenosine 5'-phosphosulfate metabolic process | 0.005311093 | 0.048129721 | SLC26A1/SLC26A2 |
| GO:1902993 | positive regulation of amyloid precursor protein catabolic process | 0.005311093 | 0.048129721 | ABCA2/SLC2A13 |

**Supplementary Table-S6.** The Gene Ontology analysis indicates notable enrichments in the cellular components (CC) among differentially methylated genes.

| **ID** | **Description** | **P-value** | **FDR** | **Gene id** |
| --- | --- | --- | --- | --- |
| GO:0016324 | Apical plasma membrane | 5.44191E-18 | 8.43496E-16 | ABCC2/CFTR/PRKAA1/PRKCI/PRKCZ/SLC11A2/SLC15A2/SLC16A3/SLC23A2/SLC26A2/SLC2A2/SLC2A9/SLC34A2/SLC39A4/SLC4A11/SLC4A2/SLC5A6/SLC6A18/SLC6A20/SLC6A6/SLC9A1 |
| GO:0045177 | Apical part of cell | 1.39749E-17 | 1.08305E-15 | ABCC2/CFTR/PRKAA1/PRKCI/PRKCZ/SLC11A2/SLC15A2/SLC16A3/SLC23A2/SLC25A27/SLC26A2/SLC2A2/SLC2A9/SLC34A2/SLC39A4/SLC4A11/SLC4A2/SLC5A6/SLC6A18/SLC6A20/SLC6A6/SLC9A1 |
| GO:0016323 | Basolateral plasma membrane | 9.23378E-10 | 4.77078E-08 | SLC13A3/SLC16A3/SLC1A3/SLC23A2/SLC26A1/SLC2A9/SLC4A11/SLC4A2/SLC5A6/SLC6A6/SLC9A1/SLCO1B3 |
| GO:0031526 | Brush border membrane | 2.18667E-07 | 8.47335E-06 | ABCC2/SLC11A2/SLC28A3/SLC34A2/SLC5A6/SLC6A18 |
| GO:0005903 | Brush border | 4.60131E-07 | 1.4264E-05 | ABCC2/SLC11A2/SLC28A3/SLC2A2/SLC34A2/SLC5A6/SLC6A18 |
| GO:0098862 | Cluster of actin-based cell projections | 6.96323E-06 | 0.000179884 | ABCC2/SLC11A2/SLC28A3/SLC2A2/SLC34A2/SLC5A6/SLC6A18 |
| GO:0031301 | Integral component of organelle membrane | 3.6966E-05 | 0.000818532 | ABCB10/SLC17A7/SLC25A3/SLC30A3/SLC35B4/SLC37A2/SLC38A9/SLC6A17/TAP2 |
| GO:0005743 | Mitochondrial inner membrane | 6.22107E-05 | 0.001190399 | ABCB10/SLC25A13/SLC25A16/SLC25A24/SLC25A26/SLC25A27/SLC25A3/SLC25A35/SLC25A36/SLC25A37 |
| GO:0031300 | Intrinsic component of organelle membrane | 6.91199E-05 | 0.001190399 | ABCB10/SLC17A7/SLC25A3/SLC30A3/SLC35B4/SLC37A2/SLC38A9/SLC6A17/TAP2 |
| GO:0031253 | Cell projection membrane | 0.000147425 | 0.002265877 | ABCC2/SLC11A2/SLC26A2/SLC28A3/SLC34A2/SLC5A6/SLC6A18/SLC6A6 |
| GO:0098533 | Atpase dependent transmembrane transport complex | 0.000160804 | 0.002265877 | ABCA2/ABCC9/SLC9A1 |
| GO:0045178 | Basal part of cell | 0.000253236 | 0.002932056 | SLC11A2/SLC1A3/SLC23A2/SLC5A6 |
| GO:0005765 | Lysosomal membrane | 0.000264831 | 0.002932056 | ABCA2/CFTR/SLC11A2/SLC17A5/SLC30A3/SLC30A4/SLC38A9/SLC44A2 |
| GO:0098852 | Lytic vacuole membrane | 0.000264831 | 0.002932056 | ABCA2/CFTR/SLC11A2/SLC17A5/SLC30A3/SLC30A4/SLC38A9/SLC44A2 |
| GO:0030285 | Integral component of synaptic vesicle membrane | 0.000460393 | 0.00475739 | SLC17A7/SLC30A3/SLC6A17 |
| GO:0005774 | Vacuolar membrane | 0.000626735 | 0.006071491 | ABCA2/CFTR/SLC11A2/SLC17A5/SLC30A3/SLC30A4/SLC38A9/SLC44A2 |
| GO:0098563 | Intrinsic component of synaptic vesicle membrane | 0.001196621 | 0.009764802 | SLC17A7/SLC30A3/SLC6A17 |
| GO:0030672 | Synaptic vesicle membrane | 0.001196976 | 0.009764802 | SLC17A5/SLC17A7/SLC30A3/SLC6A17 |
| GO:0099501 | Exocytic vesicle membrane | 0.001196976 | 0.009764802 | SLC17A5/SLC17A7/SLC30A3/SLC6A17 |
| GO:0009925 | Basal plasma membrane | 0.001517277 | 0.011758897 | SLC1A3/SLC23A2/SLC5A6 |
| GO:0090533 | Cation-transporting atpase complex | 0.002857092 | 0.021088061 | ABCC9/SLC9A1 |
| GO:0031902 | Late endosome membrane | 0.003536134 | 0.024913674 | SLC11A2/SLC30A3/SLC30A4/SLC38A9 |
| GO:0031528 | Microvillus membrane | 0.005930606 | 0.039967125 | SLC26A2/SLC6A6 |

**Supplementary Table-S7.** The Gene Ontology analysis suggests that Molecular functions (MF) primarily entail active transport activities.

| **ID** | **Description** | **p-value** | **FDR** | **Gene ID** |
| --- | --- | --- | --- | --- |
| GO:0022804 | Active transmembrane transporter activity | 6.8327E-69 | 1.46903E-66 | ABCA1/ABCA12/ABCA2/ABCB10/ABCB5/ABCC12/ABCC2/ABCC9/CFTR/SLC11A2/SLC12A5/SLC12A7/SLC12A9/SLC13A3/SLC15A2/SLC16A11/SLC16A3/SLC17A5/SLC17A7/SLC1A3/SLC1A7/SLC22A3/SLC23A2/SLC24A5/SLC25A16/SLC25A3/SLC26A1/SLC26A2/SLC28A3/SLC2A13/SLC2A9/SLC30A3/SLC33A1/SLC34A2/SLC37A2/SLC45A4/SLC4A11/SLC4A2/SLC4A3/SLC5A6/SLC6A12/SLC6A15/SLC6A17/SLC6A18/SLC6A20/SLC6A6/SLC9A1/SLC9A5/SLCO1B3/SLCO2A1/SLCO5A1/TAP2 |
| GO:0015291 | Secondary active transmembrane transporter activity | 1.56709E-55 | 1.68462E-53 | SLC11A2/SLC12A5/SLC12A7/SLC12A9/SLC13A3/SLC15A2/SLC16A11/SLC16A3/SLC17A5/SLC17A7/SLC1A3/SLC1A7/SLC22A3/SLC23A2/SLC24A5/SLC25A16/SLC25A3/SLC26A1/SLC26A2/SLC28A3/SLC2A13/SLC2A9/SLC33A1/SLC34A2/SLC37A2/SLC45A4/SLC4A11/SLC4A2/SLC4A3/SLC5A6/SLC6A12/SLC6A15/SLC6A17/SLC6A18/SLC6A20/SLC6A6/SLC9A1/SLC9A5/SLCO1B3/SLCO2A1/SLCO5A1 |
| GO:0008509 | Anion transmembrane transporter activity | 8.67007E-55 | 6.21355E-53 | ABCC2/CFTR/SLC12A5/SLC12A7/SLC12A9/SLC13A3/SLC16A11/SLC16A3/SLC17A5/SLC17A7/SLC19A2/SLC1A3/SLC1A7/SLC22A3/SLC23A2/SLC24A5/SLC25A13/SLC25A16/SLC25A24/SLC25A36/SLC25A44/SLC26A1/SLC26A2/SLC2A2/SLC2A9/SLC33A1/SLC35B4/SLC37A2/SLC38A9/SLC43A1/SLC43A2/SLC4A11/SLC4A2/SLC4A3/SLC5A6/SLC6A12/SLC6A15/SLC6A18/SLC6A20/SLC6A6/SLC9A1/SLC9A5/SLCO1B3/SLCO2A1/SLCO5A1 |
| GO:0008514 | Organic anion transmembrane transporter activity | 4.7295E-50 | 2.54211E-48 | ABCC2/CFTR/SLC13A3/SLC16A11/SLC16A3/SLC17A5/SLC17A7/SLC19A2/SLC1A3/SLC1A7/SLC22A3/SLC23A2/SLC25A13/SLC25A16/SLC25A24/SLC25A36/SLC25A44/SLC26A1/SLC26A2/SLC2A2/SLC2A9/SLC33A1/SLC35B4/SLC37A2/SLC38A9/SLC43A1/SLC43A2/SLC4A11/SLC4A3/SLC5A6/SLC6A12/SLC6A15/SLC6A18/SLC6A20/SLC6A6/SLCO1B3/SLCO2A1/SLCO5A1 |
| GO:0022853 | Active ion transmembrane transporter activity | 1.73926E-48 | 7.4788E-47 | ABCC9/SLC11A2/SLC12A5/SLC12A7/SLC12A9/SLC13A3/SLC15A2/SLC17A5/SLC17A7/SLC1A3/SLC22A3/SLC23A2/SLC24A5/SLC25A3/SLC26A1/SLC26A2/SLC28A3/SLC2A13/SLC2A9/SLC30A3/SLC33A1/SLC34A2/SLC37A2/SLC45A4/SLC4A11/SLC4A2/SLC4A3/SLC5A6/SLC6A12/SLC6A15/SLC6A20/SLC6A6/SLC9A1/SLC9A5/SLCO1B3/SLCO2A1/SLCO5A1 |
| GO:0015293 | Symporter activity | 1.30769E-42 | 4.68588E-41 | SLC11A2/SLC12A5/SLC12A7/SLC12A9/SLC13A3/SLC15A2/SLC16A11/SLC16A3/SLC17A5/SLC17A7/SLC1A3/SLC1A7/SLC22A3/SLC23A2/SLC24A5/SLC25A3/SLC28A3/SLC2A13/SLC2A9/SLC33A1/SLC34A2/SLC45A4/SLC4A11/SLC5A6/SLC6A12/SLC6A15/SLC6A17/SLC6A18/SLC6A20/SLC6A6 |
| GO:0015294 | Solute:cation symporter activity | 9.00528E-34 | 2.76591E-32 | SLC11A2/SLC12A5/SLC12A7/SLC12A9/SLC13A3/SLC15A2/SLC17A5/SLC17A7/SLC1A3/SLC22A3/SLC23A2/SLC25A3/SLC28A3/SLC2A13/SLC2A9/SLC33A1/SLC34A2/SLC45A4/SLC5A6/SLC6A12/SLC6A15/SLC6A20/SLC6A6 |
| GO:0046943 | Carboxylic acid transmembrane transporter activity | 9.02616E-32 | 2.42578E-30 | ABCC2/SLC13A3/SLC16A11/SLC16A3/SLC17A5/SLC17A7/SLC19A2/SLC1A3/SLC1A7/SLC23A2/SLC25A13/SLC25A44/SLC26A1/SLC26A2/SLC38A9/SLC43A1/SLC43A2/SLC5A6/SLC6A12/SLC6A15/SLC6A18/SLC6A20/SLC6A6/SLCO1B3/SLCO2A1 |
| GO:0005342 | Organic acid transmembrane transporter activity | 1.06276E-31 | 2.5388E-30 | ABCC2/SLC13A3/SLC16A11/SLC16A3/SLC17A5/SLC17A7/SLC19A2/SLC1A3/SLC1A7/SLC23A2/SLC25A13/SLC25A44/SLC26A1/SLC26A2/SLC38A9/SLC43A1/SLC43A2/SLC5A6/SLC6A12/SLC6A15/SLC6A18/SLC6A20/SLC6A6/SLCO1B3/SLCO2A1 |
| GO:0015077 | Monovalent inorganic cation transmembrane transporter activity | 2.20208E-26 | 4.73448E-25 | ABCC9/SLC11A2/SLC12A5/SLC12A7/SLC12A9/SLC13A3/SLC15A2/SLC17A5/SLC17A7/SLC1A3/SLC22A3/SLC23A2/SLC24A5/SLC25A3/SLC28A3/SLC2A13/SLC2A9/SLC33A1/SLC34A2/SLC45A4/SLC4A11/SLC5A6/SLC6A12/SLC6A15/SLC6A20/SLC6A6/SLC9A1/SLC9A5 |
| GO:0046873 | Metal ion transmembrane transporter activity | 1.19717E-23 | 2.33993E-22 | ABCC9/SLC11A2/SLC12A5/SLC12A7/SLC12A9/SLC13A3/SLC17A7/SLC1A3/SLC22A3/SLC23A2/SLC24A5/SLC25A37/SLC28A3/SLC30A3/SLC30A4/SLC34A2/SLC39A10/SLC39A4/SLC39A9/SLC4A11/SLC5A6/SLC6A12/SLC6A15/SLC6A20/SLC6A6/SLC9A1/SLC9A5 |
| GO:0015081 | Sodium ion transmembrane transporter activity | 1.05686E-17 | 1.89353E-16 | SLC13A3/SLC17A7/SLC1A3/SLC22A3/SLC23A2/SLC24A5/SLC28A3/SLC34A2/SLC4A11/SLC5A6/SLC6A12/SLC6A15/SLC6A20/SLC6A6/SLC9A1/SLC9A5 |
| GO:0015171 | Amino acid transmembrane transporter activity | 5.97921E-17 | 9.88869E-16 | SLC17A7/SLC1A3/SLC1A7/SLC25A13/SLC25A44/SLC38A9/SLC43A1/SLC43A2/SLC6A12/SLC6A15/SLC6A18/SLC6A20/SLC6A6 |
| GO:0015370 | Solute:sodium symporter activity | 5.33743E-16 | 8.19676E-15 | SLC13A3/SLC17A7/SLC1A3/SLC22A3/SLC23A2/SLC28A3/SLC34A2/SLC5A6/SLC6A12/SLC6A15/SLC6A20/SLC6A6 |
| GO:0015295 | Solute:proton symporter activity | 1.09558E-14 | 1.57033E-13 | SLC11A2/SLC15A2/SLC17A5/SLC25A3/SLC2A13/SLC2A9/SLC33A1/SLC45A4 |
| GO:0005310 | Dicarboxylic acid transmembrane transporter activity | 1.20104E-13 | 1.6139E-12 | ABCC2/SLC13A3/SLC17A7/SLC19A2/SLC1A3/SLC1A7/SLC25A13/SLC26A1/SLC26A2 |
| GO:1901505 | Carbohydrate derivative transmembrane transporter activity | 1.95882E-13 | 2.47733E-12 | SLC17A5/SLC25A13/SLC25A16/SLC25A24/SLC25A36/SLC25A44/SLC28A3/SLC33A1/SLC35B4/SLC37A2 |
| GO:1901682 | Sulfur compound transmembrane transporter activity | 2.49894E-13 | 2.98485E-12 | SLC13A3/SLC19A2/SLC19A3/SLC25A26/SLC26A1/SLC26A2/SLC33A1/SLC5A6/SLC6A6 |
| GO:0042626 | Atpase-coupled transmembrane transporter activity | 1.09351E-12 | 1.23739E-11 | ABCA1/ABCA12/ABCA2/ABCB10/ABCB5/ABCC12/ABCC2/ABCC9/CFTR/SLC30A3/TAP2 |
| GO:0015399 | Primary active transmembrane transporter activity | 2.33092E-12 | 2.50574E-11 | ABCA1/ABCA12/ABCA2/ABCB10/ABCB5/ABCC12/ABCC2/ABCC9/CFTR/SLC30A3/TAP2 |
| GO:0015103 | Inorganic anion transmembrane transporter activity | 2.99771E-12 | 3.06909E-11 | CFTR/SLC12A5/SLC12A7/SLC12A9/SLC17A7/SLC22A3/SLC26A1/SLC26A2/SLC37A2/SLC4A11/SLC4A2/SLC4A3 |
| GO:0015297 | Antiporter activity | 4.8674E-12 | 4.75678E-11 | SLC24A5/SLC25A16/SLC26A1/SLC26A2/SLC37A2/SLC4A11/SLC4A2/SLC4A3/SLC9A1/SLC9A5 |
| GO:0015078 | Proton transmembrane transporter activity | 1.5535E-11 | 1.45218E-10 | SLC11A2/SLC15A2/SLC17A5/SLC25A3/SLC2A13/SLC2A9/SLC33A1/SLC45A4/SLC4A11/SLC9A1/SLC9A5 |
| GO:0004697 | Protein kinase C activity | 6.36629E-11 | 5.47501E-10 | PRKCA/PRKCB/PRKCH/PRKCI/PRKCQ/PRKCZ |
| GO:0004698 | Calcium-dependent protein kinase C activity | 6.36629E-11 | 5.47501E-10 | PRKCA/PRKCB/PRKCH/PRKCI/PRKCQ/PRKCZ |
| GO:0005343 | Organic acid:sodium symporter activity | 6.63765E-11 | 5.48882E-10 | SLC13A3/SLC1A3/SLC23A2/SLC6A12/SLC6A15/SLC6A20/SLC6A6 |
| GO:0015932 | Nucleobase-containing compound transmembrane transporter activity | 1.32321E-10 | 1.05367E-09 | SLC25A13/SLC25A16/SLC25A24/SLC25A36/SLC25A44/SLC28A3/SLC33A1/SLC35B4 |
| GO:0015179 | L-amino acid transmembrane transporter activity | 2.76784E-10 | 2.1253E-09 | SLC17A7/SLC1A3/SLC1A7/SLC25A13/SLC38A9/SLC43A1/SLC43A2/SLC6A20 |
| GO:0015605 | Organophosphate ester transmembrane transporter activity | 3.99394E-10 | 2.96102E-09 | SLC25A13/SLC25A16/SLC25A24/SLC25A36/SLC25A44/SLC33A1/SLC37A2 |
| GO:0009931 | Calcium-dependent protein serine/threonine kinase activity | 7.82022E-10 | 5.60449E-09 | PRKCA/PRKCB/PRKCH/PRKCI/PRKCQ/PRKCZ |
| GO:0010857 | Calcium-dependent protein kinase activity | 1.03885E-09 | 7.20492E-09 | PRKCA/PRKCB/PRKCH/PRKCI/PRKCQ/PRKCZ |
| GO:0015301 | Anion:anion antiporter activity | 2.86512E-09 | 1.86667E-08 | SLC26A1/SLC26A2/SLC37A2/SLC4A11/SLC4A2/SLC4A3 |
| GO:0140323 | Solute:anion antiporter activity | 2.86512E-09 | 1.86667E-08 | SLC26A1/SLC26A2/SLC37A2/SLC4A11/SLC4A2/SLC4A3 |
| GO:0042887 | Amide transmembrane transporter activity | 3.49071E-09 | 2.20736E-08 | ABCA1/SLC13A3/SLC15A2/SLC19A2/SLC33A1/SLC5A6/TAP2 |
| GO:0000295 | Adenine nucleotide transmembrane transporter activity | 4.48242E-09 | 2.677E-08 | SLC25A13/SLC25A16/SLC25A24/SLC25A36/SLC25A44/SLC33A1 |
| GO:0005346 | Purine ribonucleotide transmembrane transporter activity | 4.48242E-09 | 2.677E-08 | SLC25A13/SLC25A16/SLC25A24/SLC25A36/SLC25A44/SLC33A1 |
| GO:0015216 | Purine nucleotide transmembrane transporter activity | 5.5377E-09 | 3.21785E-08 | SLC25A13/SLC25A16/SLC25A24/SLC25A36/SLC25A44/SLC33A1 |
| GO:0015215 | Nucleotide transmembrane transporter activity | 8.26889E-09 | 4.67845E-08 | SLC25A13/SLC25A16/SLC25A24/SLC25A36/SLC25A44/SLC33A1 |
| GO:0008028 | Monocarboxylic acid transmembrane transporter activity | 9.98391E-09 | 5.50395E-08 | SLC16A11/SLC16A3/SLC5A6/SLC6A12/SLC6A6/SLCO1B3/SLCO2A1 |
| GO:0046915 | Transition metal ion transmembrane transporter activity | 1.71018E-08 | 9.1922E-08 | SLC11A2/SLC25A37/SLC30A3/SLC30A4/SLC39A10/SLC39A4 |
| GO:0015108 | Chloride transmembrane transporter activity | 2.33077E-08 | 1.22223E-07 | CFTR/SLC12A5/SLC12A7/SLC12A9/SLC17A7/SLC22A3/SLC26A1/SLC26A2 |
| GO:0015106 | Bicarbonate transmembrane transporter activity | 3.63207E-08 | 1.85927E-07 | CFTR/SLC26A1/SLC26A2/SLC4A11/SLC4A3 |
| GO:0005347 | ATP transmembrane transporter activity | 1.15294E-07 | 5.76472E-07 | SLC25A13/SLC25A16/SLC25A24/SLC25A36/SLC25A44 |
| GO:0090482 | Vitamin transmembrane transporter activity | 2.05887E-07 | 1.00604E-06 | SLC19A2/SLC19A3/SLC23A2/SLC2A2/SLC5A6 |
| GO:0005313 | L-glutamate transmembrane transporter activity | 4.14071E-07 | 1.97834E-06 | SLC17A7/SLC1A3/SLC1A7/SLC25A13 |
| GO:0015175 | Neutral amino acid transmembrane transporter activity | 4.73728E-07 | 2.21417E-06 | SLC38A9/SLC43A1/SLC43A2/SLC6A20/SLC6A6 |
| GO:0005283 | Amino acid:sodium symporter activity | 5.6265E-07 | 2.57382E-06 | SLC1A3/SLC6A12/SLC6A15/SLC6A20 |
| GO:0015144 | Carbohydrate transmembrane transporter activity | 7.34065E-07 | 3.21448E-06 | SLC17A5/SLC23A2/SLC2A2/SLC2A9/SLC45A4 |
| GO:0005402 | Carbohydrate:cation symporter activity | 7.47553E-07 | 3.21448E-06 | SLC17A5/SLC23A2/SLC2A9/SLC45A4 |
| GO:0015172 | Acidic amino acid transmembrane transporter activity | 7.47553E-07 | 3.21448E-06 | SLC17A7/SLC1A3/SLC1A7/SLC25A13 |
| GO:0015377 | Cation:chloride symporter activity | 9.74121E-07 | 4.10659E-06 | SLC12A5/SLC12A7/SLC12A9/SLC22A3 |
| GO:0005416 | Amino acid:cation symporter activity | 1.57526E-06 | 6.51308E-06 | SLC1A3/SLC6A12/SLC6A15/SLC6A20 |
| GO:0072349 | Modified amino acid transmembrane transporter activity | 1.96213E-06 | 7.95957E-06 | SLC13A3/SLC19A2/SLC5A6/SLC6A20 |
| GO:0005385 | Zinc ion transmembrane transporter activity | 2.41526E-06 | 9.6163E-06 | SLC30A3/SLC30A4/SLC39A10/SLC39A4 |
| GO:0015296 | Anion:cation symporter activity | 2.94157E-06 | 1.14989E-05 | SLC12A5/SLC12A7/SLC12A9/SLC22A3 |
| GO:0016887 | Atpase activity | 3.95072E-06 | 1.51679E-05 | ABCA1/ABCA12/ABCA2/ABCB10/ABCB5/ABCC12/ABCC2/ABCC9/ABCE1/CFTR/TAP2 |
| GO:0015101 | Organic cation transmembrane transporter activity | 4.24295E-06 | 1.60041E-05 | SLC22A3/SLC44A1/SLC44A2/SLC6A20 |
| GO:0015079 | Potassium ion transmembrane transporter activity | 7.9378E-06 | 2.94246E-05 | ABCC9/SLC12A5/SLC12A7/SLC12A9/SLC24A5/SLC9A1/SLC9A5 |
| GO:0072509 | Divalent inorganic cation transmembrane transporter activity | 1.01618E-05 | 3.70301E-05 | SLC11A2/SLC24A5/SLC30A3/SLC30A4/SLC39A10/SLC39A4/SLC41A2 |
| GO:1904680 | Peptide transmembrane transporter activity | 1.22573E-05 | 4.39221E-05 | ABCA1/SLC13A3/SLC15A2/TAP2 |
| GO:0005452 | Inorganic anion exchanger activity | 2.65213E-05 | 9.34766E-05 | SLC4A11/SLC4A2/SLC4A3 |
| GO:0015347 | Sodium-independent organic anion transmembrane transporter activity | 5.14104E-05 | 0.000175448 | SLCO1B3/SLCO2A1/SLCO5A1 |
| GO:0022821 | Potassium ion antiporter activity | 5.14104E-05 | 0.000175448 | SLC24A5/SLC9A1/SLC9A5 |
| GO:0035173 | Histone kinase activity | 6.22182E-05 | 0.000209014 | PRKAA1/PRKCA/PRKCB |
| GO:1901618 | Organic hydroxy compound transmembrane transporter activity | 7.13907E-05 | 0.000236138 | SLC16A3/SLC22A3/SLC2A13/SLCO1B3 |
| GO:0005326 | Neurotransmitter transmembrane transporter activity | 8.80691E-05 | 0.000286892 | SLC17A7/SLC22A3/SLC6A15 |
| GO:0015145 | Monosaccharide transmembrane transporter activity | 0.00015882 | 0.000509646 | SLC23A2/SLC2A2/SLC2A9 |
| GO:0051119 | Sugar transmembrane transporter activity | 0.000204884 | 0.000647796 | SLC23A2/SLC2A2/SLC2A9 |
| GO:0015491 | Cation:cation antiporter activity | 0.000230835 | 0.00071927 | SLC24A5/SLC9A1/SLC9A5 |
| GO:0042910 | Xenobiotic transmembrane transporter activity | 0.000258823 | 0.000794956 | ABCC2/SLC22A3/SLC6A6 |
| GO:0005319 | Lipid transporter activity | 0.000395661 | 0.001198128 | ABCA1/ABCA12/ABCA2/SLCO1B3/SLCO2A1 |
| GO:0015298 | Solute:cation antiporter activity | 0.000431622 | 0.00128887 | SLC24A5/SLC9A1/SLC9A5 |
| GO:0005381 | Iron ion transmembrane transporter activity | 0.000931295 | 0.002669714 | SLC11A2/SLC25A37 |
| GO:0015651 | Quaternary ammonium group transmembrane transporter activity | 0.000931295 | 0.002669714 | SLC22A3/SLC6A20 |
| GO:0019531 | Oxalate transmembrane transporter activity | 0.000931295 | 0.002669714 | SLC26A1/SLC26A2 |
| GO:0005436 | Sodium:phosphate symporter activity | 0.001134827 | 0.003088453 | SLC17A7/SLC34A2 |
| GO:0015386 | Potassium:proton antiporter activity | 0.001134827 | 0.003088453 | SLC9A1/SLC9A5 |
| GO:0140326 | Atpase-coupled intramembrane lipid transporter activity | 0.001134827 | 0.003088453 | ABCA1/ABCA2 |
| GO:1901474 | Azole transmembrane transporter activity | 0.001134827 | 0.003088453 | SLC19A2/SLC19A3 |
| GO:0015385 | Sodium:proton antiporter activity | 0.001357698 | 0.003648815 | SLC9A1/SLC9A5 |
| GO:0005451 | Monovalent cation:proton antiporter activity | 0.001599731 | 0.004194416 | SLC9A1/SLC9A5 |
| GO:0008271 | Secondary active sulfate transmembrane transporter activity | 0.001599731 | 0.004194416 | SLC26A1/SLC26A2 |
| GO:0046624 | Sphingolipid transporter activity | 0.001860746 | 0.004762622 | ABCA1/ABCA2 |
| GO:0051139 | Metal ion:proton antiporter activity | 0.001860746 | 0.004762622 | SLC9A1/SLC9A5 |
| GO:0099106 | Ion channel regulator activity | 0.002271159 | 0.005744695 | ABCC9/CFTR/PRKCB/PRKCZ |
| GO:0015556 | C4-dicarboxylate transmembrane transporter activity | 0.002439017 | 0.006097543 | SLC13A3/SLC25A13 |
| GO:0015116 | Sulfate transmembrane transporter activity | 0.002755924 | 0.006810616 | SLC26A1/SLC26A2 |
| GO:0099095 | Ligand-gated anion channel activity | 0.003444412 | 0.008415324 | CFTR/SLC17A7 |
| GO:0015299 | Solute:proton antiporter activity | 0.00381565 | 0.009217582 | SLC9A1/SLC9A5 |
| GO:0004674 | Protein serine/threonine kinase activity | 0.004039706 | 0.009650408 | PRKAA1/PRKCA/PRKCB/PRKCH/PRKCI/PRKCQ/PRKCZ |
| GO:0005355 | Glucose transmembrane transporter activity | 0.004204658 | 0.009720447 | SLC2A2/SLC2A9 |
| GO:0015149 | Hexose transmembrane transporter activity | 0.004204658 | 0.009720447 | SLC2A2/SLC2A9 |
| GO:0140303 | Intramembrane lipid transporter activity | 0.004204658 | 0.009720447 | ABCA1/ABCA2 |
| GO:0016247 | Channel regulator activity | 0.004960881 | 0.011346695 | ABCC9/CFTR/PRKCB/PRKCZ |

**Supplementary Table-S8.** Gene ontology enrichment analysis and KEGG pathway analysis

| **ID** | **Description** | **p-value** | **FDR** | **Gene ID** |
| --- | --- | --- | --- | --- |
| hsa02010 | ABC transporters | 1.79033E-14 | 2.36324E-12 | ABCA1/ABCA12/ABCA2/ABCB10/ABCB5/ABCC12/  ABCC2/ABCC9/CFTR/TAP2 |
| hsa04971 | Gastric acid secretion | 4.83993E-05 | 0.003194351 | CFTR/PRKCA/PRKCB/SLC4A2/SLC9A1 |
| hsa04976 | Bile secretion | 0.000103285 | 0.004544556 | ABCC2/CFTR/SLC4A2/SLC9A1/SLCO1B3 |
| hsa05231 | Choline metabolism in cancer | 0.000163073 | 0.005044244 | PRKCA/PRKCB/SLC22A3/SLC44A1/SLC44A2 |
| hsa04972 | Pancreatic secretion | 0.000196866 | 0.005044244 | CFTR/PRKCA/PRKCB/SLC4A2/SLC9A1 |
| hsa04931 | Insulin resistance | 0.000257285 | 0.005044244 | PRKAA1/PRKCB/PRKCQ/PRKCZ/SLC2A2 |
| hsa04977 | Vitamin digestion and absorption | 0.000267498 | 0.005044244 | SLC19A2/SLC19A3/SLC5A6 |
| hsa04724 | Glutamatergic synapse | 0.000330894 | 0.005459744 | PRKCA/PRKCB/SLC17A7/SLC1A3/SLC1A7 |
| hsa04721 | Synaptic vesicle cycle | 0.000762665 | 0.011185749 | SLC17A7/SLC1A3/SLC1A7/SLC6A12 |
| hsa04727 | GABAergic synapse | 0.001250923 | 0.016512178 | PRKCA/PRKCB/SLC12A5/SLC6A12 |
| hsa04970 | Salivary secretion | 0.001414979 | 0.016979748 | PRKCA/PRKCB/SLC4A2/SLC9A1 |
| hsa04750 | Inflammatory mediator regulation of TRP channels | 0.001787233 | 0.019659558 | PRKCA/PRKCB/PRKCH/PRKCQ |
| hsa04978 | Mineral absorption | 0.003955062 | 0.040159089 | SLC11A2/SLC34A2/SLC39A4 |
